# Chronology and social significance of the “princely” barrow cemetery in Łęki Małe and the Central European Early Bronze Age

**DOI:** 10.1371/journal.pone.0300591

**Published:** 2024-05-20

**Authors:** Janusz Czebreszuk, Johannes Müller, Marzena Szmyt, Tomasz Goslar, Mateusz Jaeger, Iwona Hildebrandt-Radke, Jakub Niebieszczański, Barbara Gmińska-Nowak, Tomasz Ważny, Jutta Kneisel, Ben Krause-Kyora, Daniel Makowiecki, Artur Rewekant, Nadiia Kotova, Joanna Rennwanz, Hendrik Raese

**Affiliations:** 1 Faculty of Archaeology, Adam Mickiewicz University, Poznań, Poland; 2 Institute of Prehistoric and Protohistoric Archaeology, Kiel University, Kiel, Germany; 3 Archaeological Museum in Poznań, Poznań, Poland; 4 Poznań Radiocarbon Laboratory, Poznań Park of Science and Technology, Poznań, Poland; 5 Faculty of Geographical and Geological Sciences, Adam Mickiewicz University, Poznań, Poland; 6 Institute of European Culture, Adam Mickiewicz University, Poznań, Poland; 7 Institute of Geoecology and Geoinformation, Adam Mickiewicz University, Poznań, Poland; 8 Faculty of Fine Arts, Centre for Research and Conservation of Cultural Heritage, Nicolaus Copernicus University, Toruń, Poland; 9 Institute of Clinical Molecular Biology, Kiel University, Kiel, Germany; 10 Department of Environmental Archaeology and Human Paleoecology, Institute of Archaeology, Nicolaus Copernicus University, Toruń, Poland; 11 Independent Researcher, Konin, Poland; 12 Department of Bioarchaeology, Institute of Archaeology of National Academy of Sciences of Ukraine, Kyiv, Ukraine; 13 Institute of Archaeology and Ethnology, Polish Academy of Sciences, Poznań, Poland; Austrian Academy of Sciences, AUSTRIA

## Abstract

The “princely” barrows of Łęki Małe, Greater Poland are the oldest such monuments within the distribution area of Únětice societies in Central Europe. While in the Circum-Harz group and in Silesia similar rich furnished graves under mounds have appeared as single monuments as early as 1950 BC, Łęki Małe represents a chain of barrows constructed between 2150 BC and 1800 BC. Of the original 14 mounds, only four were preserved well enough that their complex biographies can now be reconstructed. They included ritual activities (before, during, and after the funeral), and also subsequent incursions, including robberies. The long lasting barrow cemetery at Łęki Małe can be linked to a nearby fortified site, Bruszczewo. Together, Łęki Małe and Bruszczewo represent a stable, socially differentiated society that existed for no less than 350–400 years. Therefore, it can be argued that the Early Bronze Age societies of Greater Poland were extremely sustainable in comparison to those of other Únětice regions.

## Introduction

**Fig 1:**
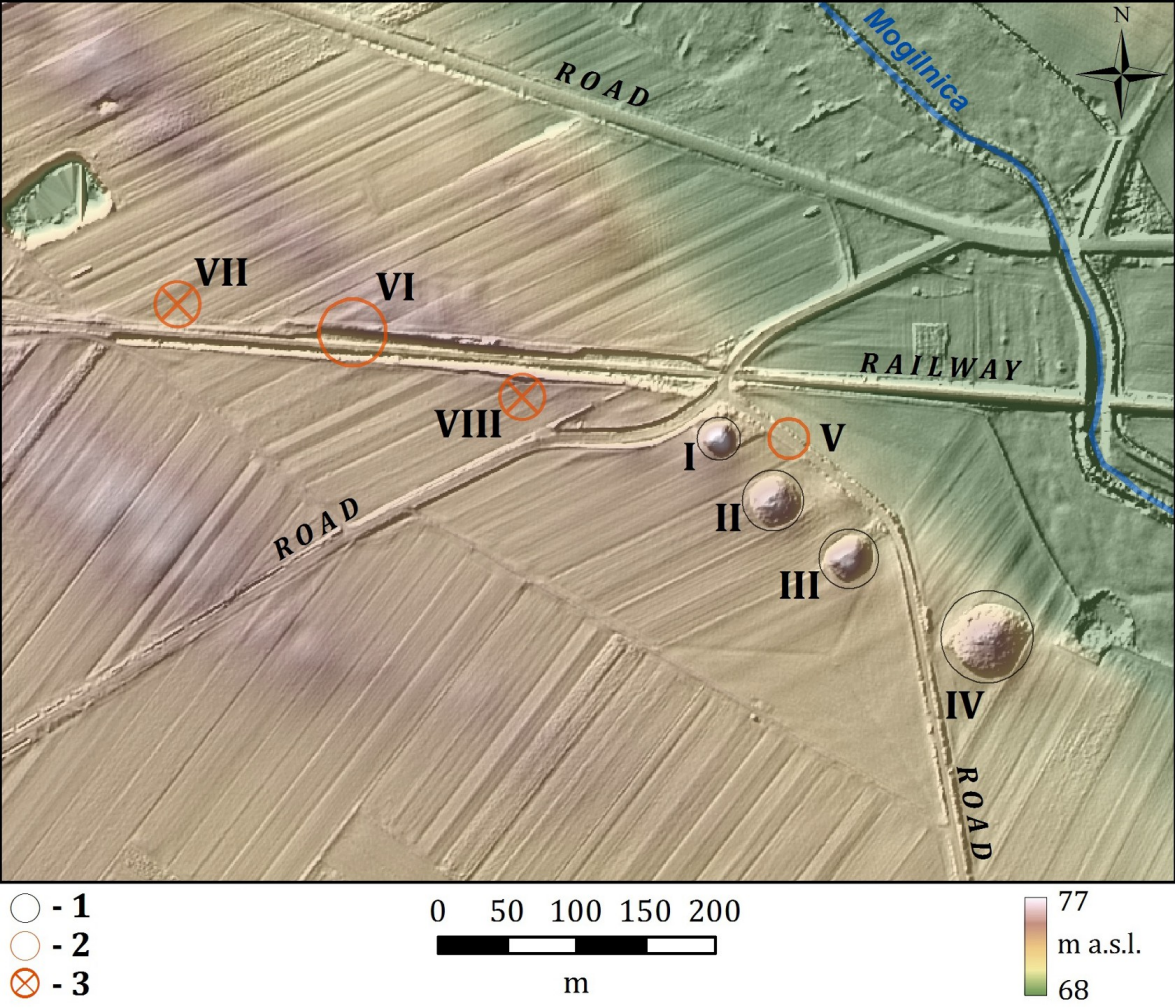
Barrow cemetery in Łęki Małe. Key: 1 –barrows excavated and then reconstructed; 2 –barrows partially preserved, 3 –barrows registered in the mid-twentieth century.

The cemetery at Łęki Małe (now Wilanowo, Kamieniec commune, Grodzisk Wielkopolski county, Greater Poland province) (Fig 1) is one of the most important sites of the Central European Early Bronze Age. It consists of large burial mounds of the Únětice phenomenon.

The barrow cemetery at Łęki Małe is a part of the Kościan group, the most northeasterly Únětice unit [1,2]. Together with the Únětice fortified settlement at Bruszczewo [3–9], it formed a regional centre during Únětice times, and provides essential information on metal production and redistribution patterns concerning the entire Kościan group.

What makes this cemetery exceptional is that it is the only site with several burial mounds, called “princely barrows” or “monumental burial mounds”, discovered so far within the Únětice distribution area [10]. At other Únětice cemetery sites, only single burial mounds were documented. This is the case of the Circum-Harz group (Bornhöck, Helmsdorf, Leubingen) and in Silesia (Kąty Wrocławskie, Szczepankowice).

The first aim of this article is to present new evidence on the chronology of the cemetery at Łęki Małe. We reconstruct the biography of the mounds by combining newest chronometric evidence (radiocarbon dating and dendrochronology) with stratigraphic information and typo-chronological considerations.

As the biography of the Łęki Małe barrows provides new insights into Únětice social, political, and religious practices and perhaps institutions, the results will be discussed in comparison with the other Únětice regions where „princely”barrows have been identified. This is our second objective: to outline the scope of necessary revisions to current models representing the functioning of the Únětice world, as revealed by multidisciplinary studies of Łęki Małe materials.

### Site background

**Fig 2:**
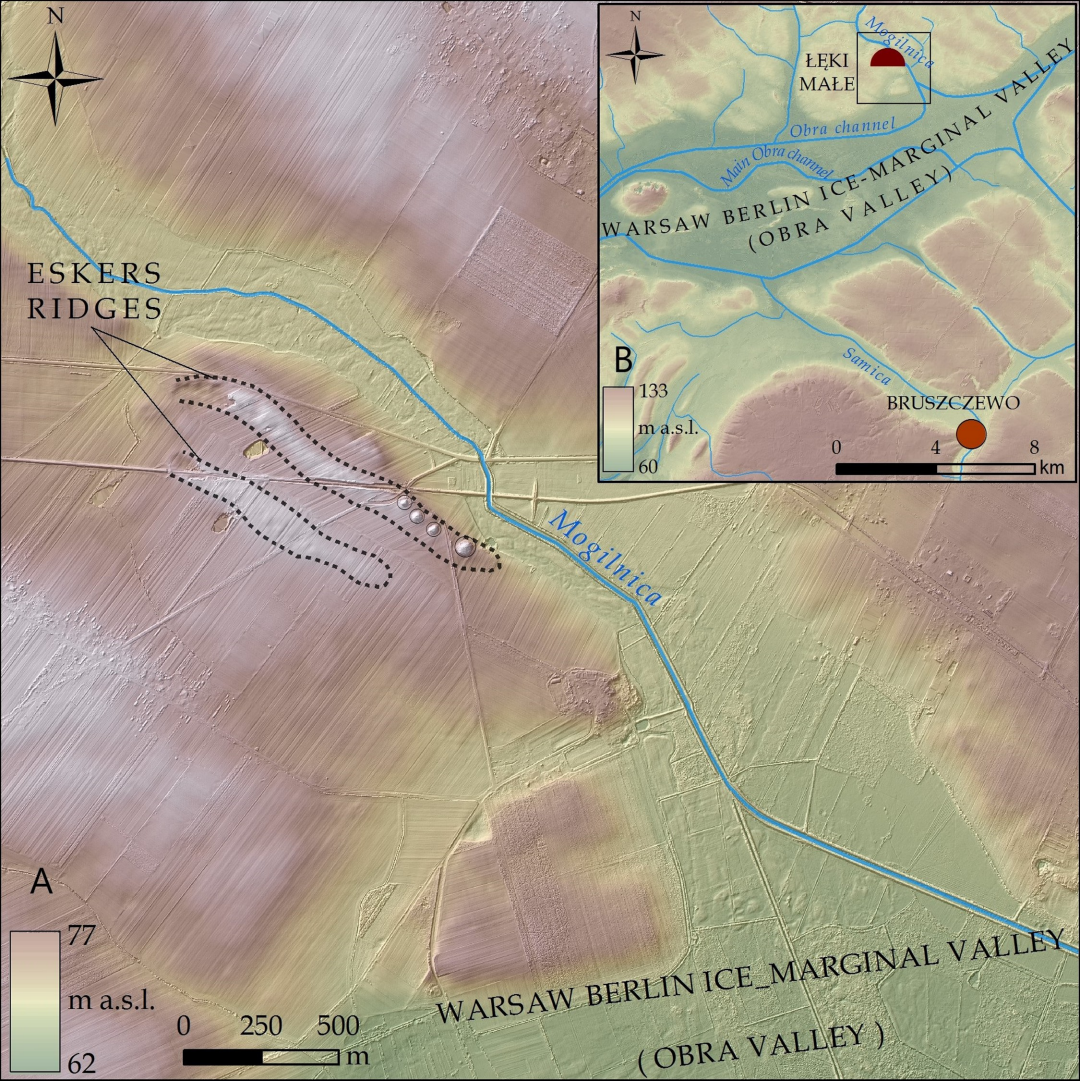
Łęki Małe. Geomorphological background of the cemetery in limited (A) and large (B) scale.

According to archival reports [11], the cemetery at Łęki Małe initially consisted of at least fourteen barrows (Fig 1 and Table 1). By the mid-20th century, nine mounds were still visible (see [10] ‐ there is no Barrow IX showed in the figure mentioned) (Fig 2). Five of these mounds were excavated in the 20th century: Barrow II ‐ partly explored in 1933 [12], Barrow I in 1953 [10], Barrow III in 1955 [13], Barrow IV in 1956–1958 [14] and finally Barrow VI in 1970 [15]. Archaeological excavation of Barrow VI in 1970 revealed it was largely destroyed in the late 19th century during construction of a local railway line.

**Table 1:** Łęki Małe. Register of barrows according to historical sources.

Barrow	References	Comments
I	[10,11]	Excavated in 1953.
II	[10–12]	Partially excavated in 1933.
III	[10,11,13]	Excavated in 1955.
IV	[10,11,14]	Excavated in 1956–1958.
V	[10,11]	Partially destroyed, cut by the road.
VI	[10,11,15]	Partly destroyed during the construction of the railway line at the end of the 19th century; cut by a railway embankment; the remains of the burial mound were excavated in 1970.
VII	[10,11]	The embankment is not marked on the map from 1954; only the location is indicated; currently invisible.
VIII	[10,11]	Partially destroyed.
IX	[10,11]	No location on the map of 1953.
X	[11]	Visible in 1881; no later information.
XI	[11]	Visible in 1881; no later information.
XII	[11]	In 1881, freshly levelled; no later information.
XIII	[11]	In 1881, freshly levelled; no later information.
XIV	[11]	In 1881, freshly levelled; no later information.

Barrow IV is situated in the southeastern-most part of the cemetery and begins the row of approx. ten barrows that stretch north-west along a minor tributary of the Obra River–Mogilnica River, whose name can be roughly translated as “the river of graves”. This burial mound is believed to be the oldest of all archaeologically excavated mounds in Łęki Małe [16].

The site where the Early Bronze cemetery was established was used several times, both before and after the period of its existence (Table 2). The earliest remains on the site are attributed to communities of the 5th millennium BC. Then, human occupations continued in the 4th and 3rd millennium BC [17]. Corded Ware communities were among the latter. Their traces are both in the form of settlement and funerary remains [18]. A grave of the Corded Ware population marks the first use of Łęki Małe for burying the dead [18,19]. Slightly later, already in the Early Bronze Age, Barrows III and IV were raised near this grave.

**Table 2:** Łęki Małe. Stages of land use in prehistory and the Middle Ages.

Stage and phase	Description	Finds	Comments	References
I	Traces of Danubian societies	Barrow IV: a pottery fragment in the embankment.	Barrow IV: a fragment of the vessel was found in the embankment (quarter IV, depth 4.15 m).	[14]
II	Settlement of the Funnel Beaker society	Barrow I: 3 pottery fragments in the embankment. Barrow III: a pit “Y”[pottery fragments, flint product, charcoal]. Barrow IV: 5 pits and material found in the embankment [ceramics, flints, daub, animal bones, charcoal, stones].	Barrow I: pottery was discovered in quarter I "on a secondary deposit" (probably in an embankment). Barrow III: the pit “Y” was located in the second quarter, under the trench “X”. Barrow IV: the pits were discovered under the embankment (in quarter I); the FBC ceramics appeared within the central tomb, and fragments of pottery and flint artefacts were also discovered in the embankment.	[10–13,17]
III.1	The Corded Ware campsite (early stage = CWC 1 in Kuyavia)	Barrow IV: pottery in the embankment.	Barrow IV: pottery was discovered in the embankment, incl. in quarter I [Inv. 1956: 351 / Cat. 1956: 1902; Inv. 1957: 371 / Cat. 1957: 1068].	[14,18]
III.2	Grave + undefined traces of the stay of the Corded Ware population (late stage = CWC 4 in Kuyavia)	Grave: vessel and stone axe. Barrow IV: pottery and flint artefacts in the embankment.	The grave was located 50 meters N from the Barrow IV and 75 meters E from the Barrow III. Barrow IV: ceramics and flint artefacts were discovered in the embankment, incl. in quarters I, III and IV [Inv. 1956: 351 / Cat. 1956: 1902).	[10,14,18]
IV	Barrow cemetery from the Early Bronze Age	Barrows I–XIV		
V	Unspecified traces of the residence of the Lusatian society	Barrow I: four pottery sherds in the embankment.	Barrow I: in the embankment (quarters III and IV).	[10,13]
VI	Undefined traces of the stay of the late La Tène population	Barrow IV: pottery in the robbery (?) trench.	Barrow IV: trench in the NW part of the embankment (3.0 x 1.0 m, 0.2 m deep).	[14]
VII	Undefined traces of the stay of the population from the period of Roman influence	Barrow III: trench "Z" [ceramics, iron slag, animal bones, charcoal].	Barrow III: trench "Z" on the NE edge of the embankment, in quarter II (1.5 x 1.1 m, 0.9 m deep).	[13]
VIII	A ritual place from the turn of the Roman period and the early Middle Ages	Numerous features located north-west of Barrow I and north of Barrows VI and VIII.		[20,21]
IX	Early Middle Ages settlement + a robbery trench (?) in the Barrow IV.	Barrow II: dugout [ceramics, iron slags]. Barrow IV: trench [ceramics]. Near Barrow IV: a pit [pottery, 2 iron knives, charcoal, animal bones].	Barrow II: a dugout at the top of the embankment. Barrow IV: a trench in the center of the barrow, reaching the bottom of the grave. Near the Barrow IV: a pit partially destroying the Corded Ware grave (see stage III.2).	[11,22]
X	Robbery trenches (?) from the Middle Ages (12th ‐ mid-13th century)	Barrow III: trenches "O" and "X" [several pottery fragments, a stone whetstone, iron slag, 2 iron crayfish, animal bones, charcoal].	Barrow III: trench "O" at the top of the embankment (9.0 x 6.8 m, depth 1.38 m); trench "X" in the quarter II of the embankment (6 x 8.5 m, 4.2 m deep).	[13]
?	Robbery trenches (?) with undefined chronology	Barrow I: trench [no information on finds]. Barrow II: unidentified disturbances [no information on finds].	Barrow I: a trench in the central part of the embankment, visible on the N-S section of the barrow. Barrow II: disturbances visible on the cross-section of the barrow, in the central part of the barrow, under the dugout dating back to the early Middle Ages.	[10,16]

The latest geoarchaeological research revealed that the selection of burial mound location was not merely inspired by a link to an earlier tradition. The cemetery was established within a specific landscape which is the middle Obra River valley. Its form shows segmentation on at least two levels. The dominant segmentation in this area consists of several tunnel valleys (Fig 2A) that cross-cut the upland perpendicularly, creating a series of upland islands [23–25]. In some places, there are accumulation structures such as eskers which formed within upland islands on the fringe of tunnel valleys. Small erosion-denudation valleys passing into tunnel valleys separate the ends of eskers from uplands; likewise, gently sloping erosive depressions give the uplands an undulating form. These erosion-denudation valleys have outlets, but in most cases are internally drained. Their northwest-southeast orientation reflects the direction of eskers and tunnel valleys, suggesting the relief may have developed due to the large amount of snowmelt water running through the marginal region of the Leszno Phase of the Vistula glaciation.

Barrows at Łęki Małe occupy the top of an esker and its extension towards the south-east: a patch of outwash plain sediments with a continuation as a moraine upland fragment (Fig 2B). In the Early Bronze Age this area was probably detached from farmland and destined exclusively to bury the deceased. From the north and south-west, some small erosion and denudation valleys that reach the Mogilnica valley cut off the area where the barrows stand. Not far away from the esker described there is another one which adjoins the former from the west, both surrounded by an outwash patch. These two esker formations, in turn, together with an outwash rise, are intersected by a shallow, northwest-southeast oriented post-glacial erosion outwash depression which also enters the Mogilnica River in its outlet section. Therefore, natural landscape boundaries separated the two esker structures along with an outwash sediment patch and a fragment of loamy upland. However, these boundaries manifested themselves with a different degree of clearness in the landscape.

**Fig 3:**
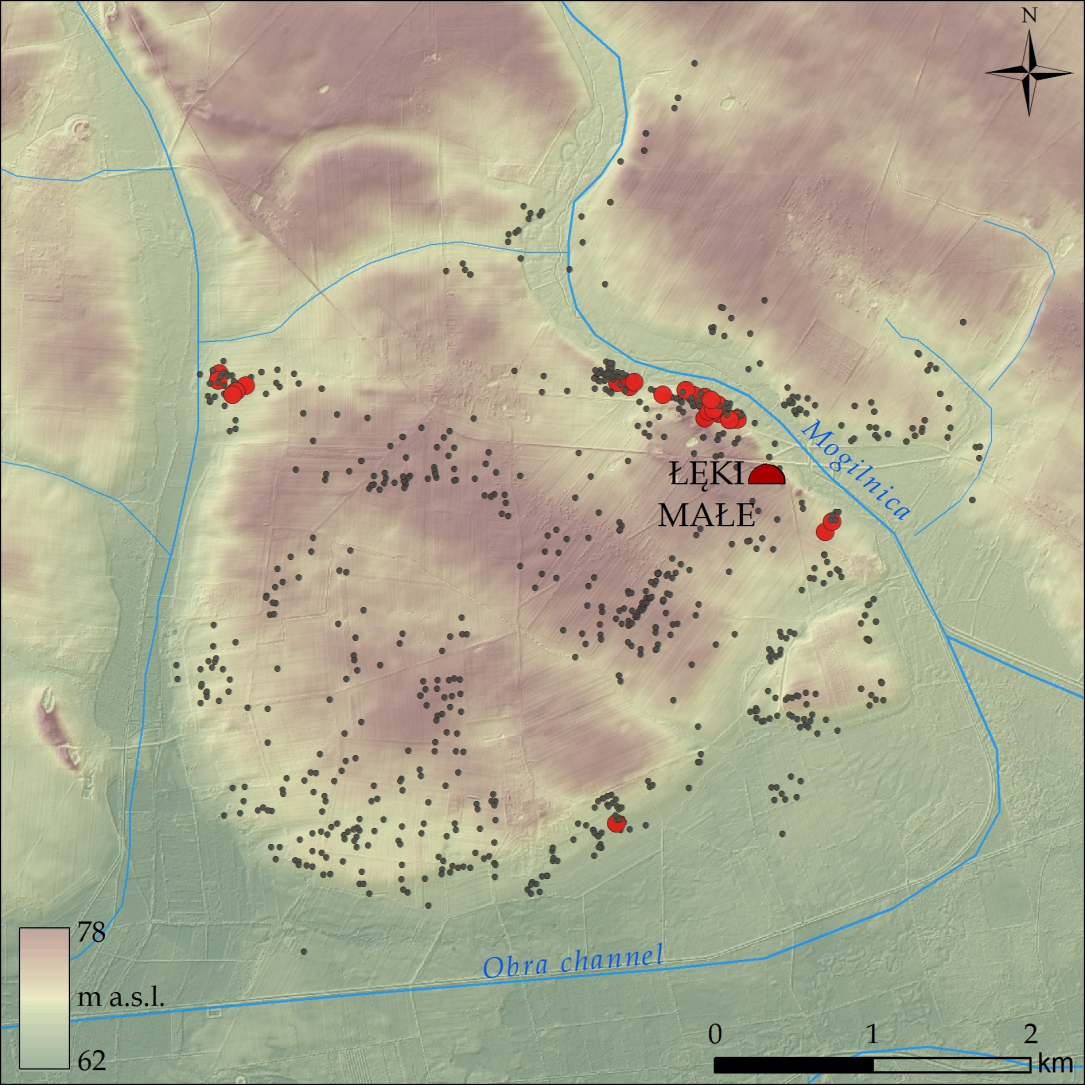
Łęki Małe. Results of field surveys around the cemetery. Key: Red dots–finds of pottery dated to the Early Bronze Age, black dots–finds of other artefacts dated to other periods of prehistory, Middle Ages or early modern times.

Field and geomagnetic surveys have shown that the investigated area ‐ except for Łęki Małe cemetery ‐ was almost free from domestic and ritual activities in the Neolithic and Bronze Age periods. Thus, we interpret the area of approx. 14 km² as a space devoid of almost all traces of human occupation in the Early Bronze Age (Fig 3).

## Methodology applied

In carrying out the tasks undertaken, we used a combination of several methods. First of all, we examined old graphic and written documentation to reconstruct stratigraphy of excavated barrows. A re-analysis of all the original drawings, plans and research diaries kept in the Scientific Archive of the Archaeological Museum in Poznań (AMP), and all printed reports [10,12–14] provided the starting point for chronometric studies based on ^14^C dating and dendrochronology.

All organic material (wood, bones, charcoal) from the Łęki Małe burial mounds stored in the AMP collection and not subjected to conservation works was considered suitable for the radiocarbon analyses. Altogether, 70 samples were submitted to the Poznań Radiocarbon Laboratory, Poland [26], of which three bone samples did not retain collagen and could not be utilized for measurement. The radiocarbon chronology was refined through replication of determinations. The improvement was also based on considerations of the age at which the person died and the limited rate of carbon exchange in the bones in the case of human remains and the age of the tree samples taken in the case of charcoal. Finally, a Bayesian approach was applied with explicit assumptions derived from stratigraphic observations. It was also possible to use results of dendrochronological analysis, which was performed on the relics of wood from Barrow I by the Laboratory of Dendrochronology in the Institute for the Study, Conservation and Restoration of Cultural Heritage of the Nicolaus Copernicus University in Toruń, Poland.

Analyses in this paper also incorporate the results of bio-archaeological research, which implicitly address chronological problems as well. The absolute chronology assessments were supported by results of the typo-chronological studies of pottery and metal artefacts.

Finally, we reconstruct the biography of the mounds by combining our new chronometric evidence with stratigraphic information and typo-chronological considerations.

### General model of barrow stratigraphy at Łęki Małe

The four best preserved barrows are numbered I to IV and provided the most comprehensive stratigraphic dataset. We have drawn information about the stratigraphy from original drawings and plans kept in the Scientific Archive of the Archaeological Museum in Poznań (hereinafter: AMP), research diaries, and printed reports [10,12–14].

For barrows no. I, III, and IV, profiles of all quarters have survived in the AMP Scientific Archive so that we can recreate two cross-cut sections. At this point, we would like to draw attention to a few stratigraphic observations that are most relevant to the following investigations.

**Fig 4:**
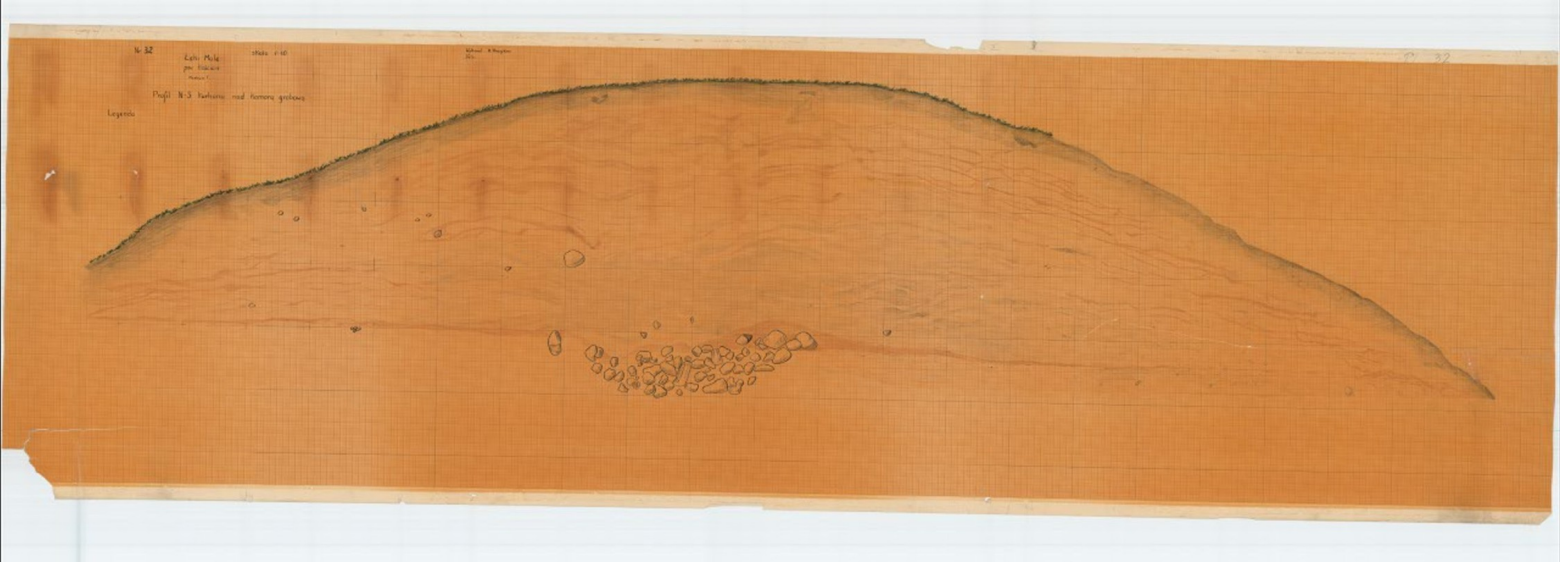
Łęki Małe. Cross-section of the Barrow I. Scientific Archive of the Archaeological Museum in Poznań.

In the case of the Barrow I, on the north-south profile, there are traces of a trench in the top part of the embankment leading to the central tomb (Fig 4), probably related to its robbery and destruction (Table 2) [10]. In Barrow III, above the stone pavement sitting in the central tombs’ place one can see curved layers of a ditch that resulted in destroying this tomb [13]. We encounter a similar case in Barrow IV; there is a distinct ditch in the uppermost central point of the structure [14]. This trench proves without a doubt that the central tomb was plundered and destroyed.

For Barrow II, we have a concise section draft (containing mainly the central part) with the stratigraphic position of main features and finds shown schematically. Moreover, several concentric hollow structures were documented [16], probably remains of a later intrusion (a robbery pit?) partially destroying the central tomb.

**Fig 5:**
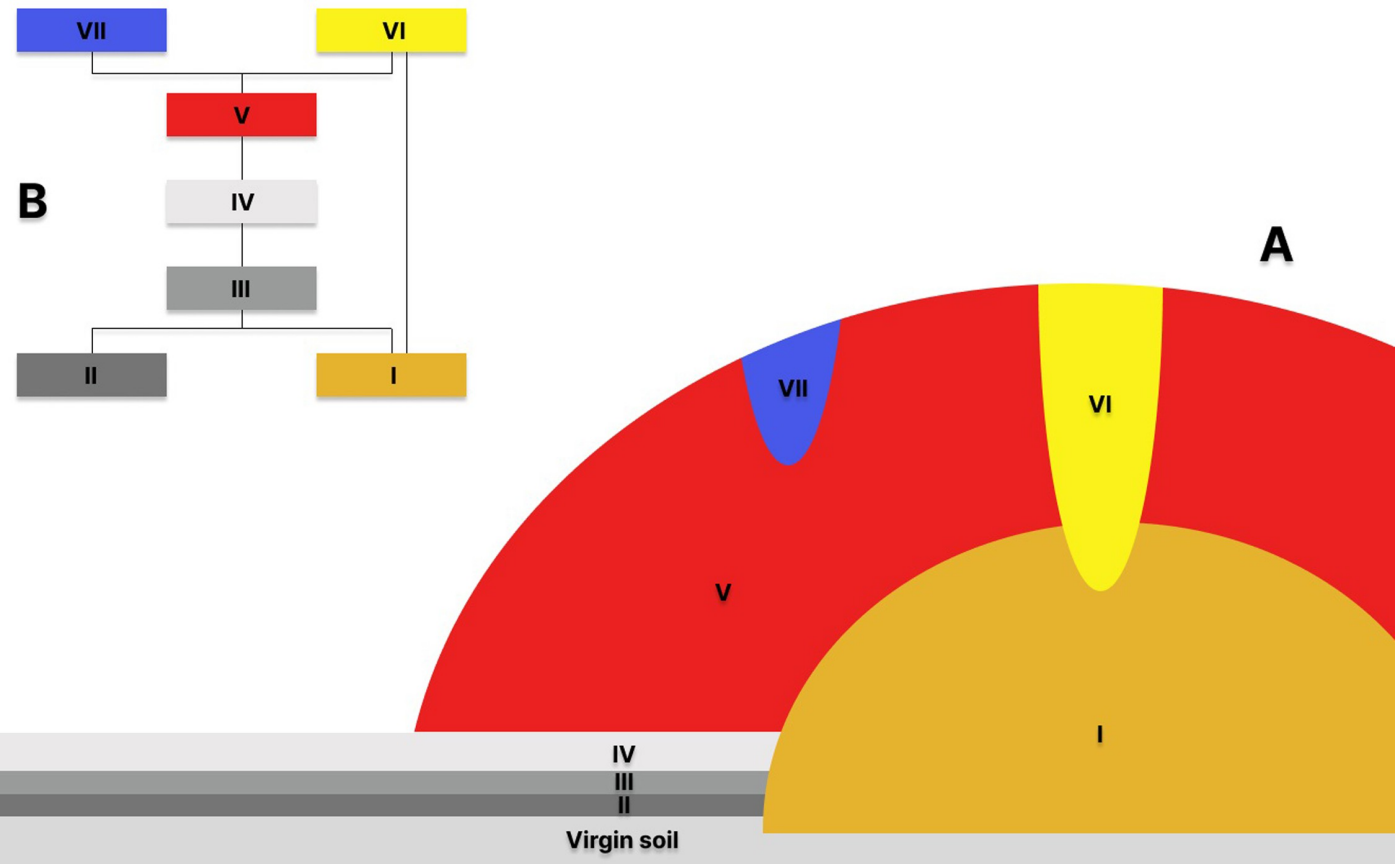
Łęki Małe. Outline of stratigraphic levels documented in the barrows (A) and Harris matrix (B).

By compiling the information about the internal structure of these four mounds, we can construct a generalized list of stratigraphic levels that were formed by similar depositional processes and in which the remains of the deceased and artefacts were deposited, and accompanying features (e.g. hearths) were located (Fig 5A):

I. In the virgin soil (central grave)
II. On the surface of the virgin soil
III. In the primary humus layer
IV. On the surface of the primary humus layer
V. In the mound (at various stages of mound construction)
VI. Destroying the central tomb
VII. Dug into the mound

In the stratigraphic levels, we were able to distinguish stratigraphic contexts, within which distinct units were identified, including: graves, hearths, accumulations of artefacts etc.

**Fig 6:**
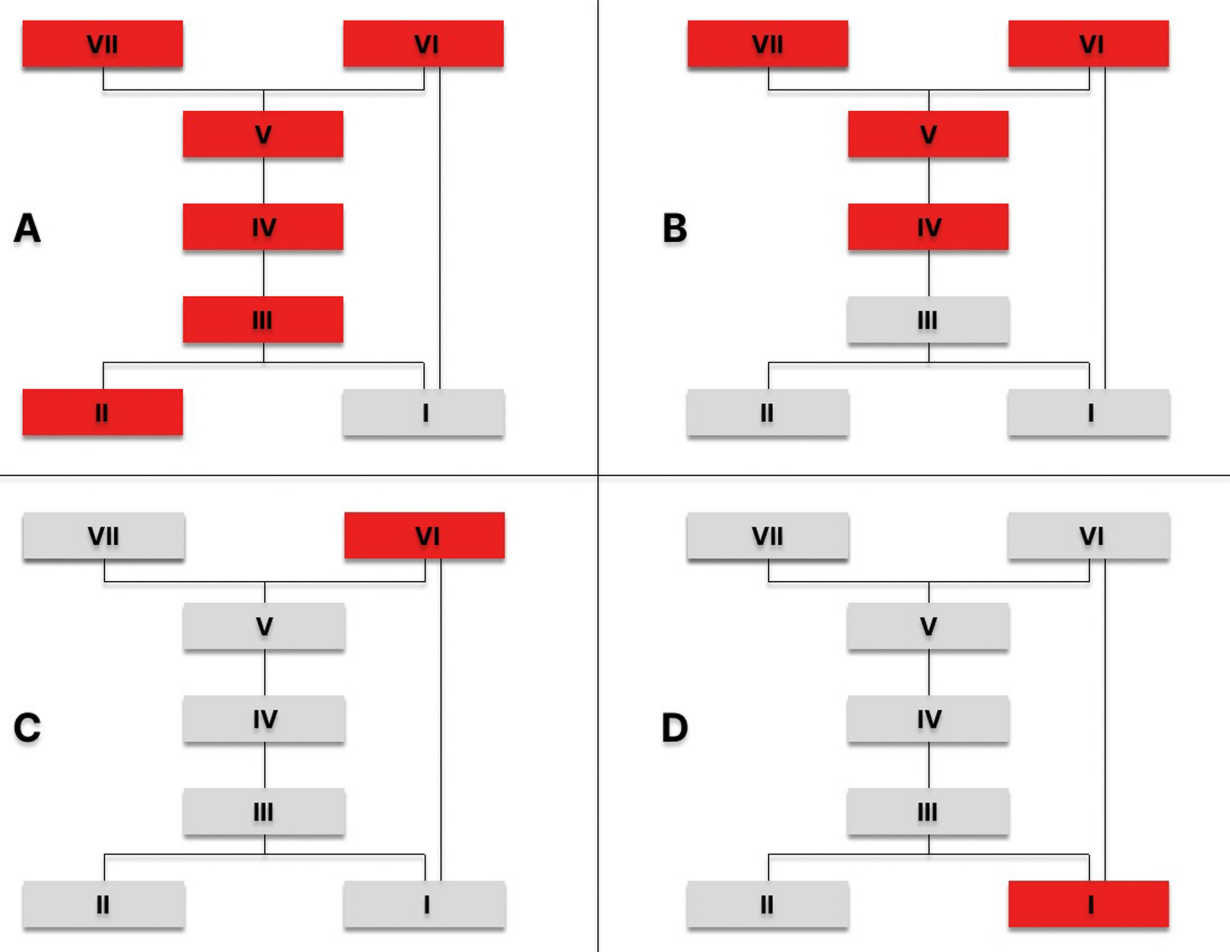
Łęki Małe. Stratigraphic levels in barrows. Red colour marks levels radiocarbon dated: A ‐ Barrow IV; B–Barrow III; C–Barrow II; D–Barrow I.

The list of stratigraphic levels can easily be transformed into the Harris matrix (Fig 5B). The categories included in the list have been used to describe the stratigraphy of each burial mound (Fig 6) and to define the stratigraphic position of dated materials.

### Special case: Clarification of bio-archaeological results, stratigraphic position and absolute chronology of side burials in Barrow IV

A certain issue which requires clarification is the composition and stratigraphic and chronological position of three side single inhumations excavated in the north-western part of the Barrow IV and named skeletons 1, 2 and 3 [14]. They were located on the surface of the virgin soil, i.e. in the stratigraphic level II (Fig 6A). According to the first anthropological report, they contained the following human remains: no. 1 ‐ a child aged 14–16, no. 2 ‐ a male aged 30–40, and no. 3 ‐ a child aged 9–10 [27].

A few years before our research (in 2013) skeleton burials no. 1, 2 and 3 were sampled for aDNA and radiocarbon analysis as part of the RISE project at the University of Gothenburg. Until now only results of examination of Burial 2 (sample RISE431) were published [28]: aDNA confirmed that it was an adult male, and ^14^C dating indicated an early chronology of the burial (OxA-27967 3762±27 BP; Table 3).

**Table 3:** Łęki Małe. List of radiocarbon dates obtained until 2015.

No.	Barrow	Stratigraphic level	Context	Unit	Sample material	Lab. no.	BP	St. deviation	Year of measurement	References
1	I	I	1x	grave A	wood	M-1325	3900	150	the 1960s	[29]
2	I	I	1x	grave A	wood	GrN-5037	3605	40	1967	[30]
3	I?	I?	1x?	grave A?	wood	Bln-1293	3620	106	1974	[31]
4	I?	I?	1x?	grave A?	humic acid from the sample Bln-1293	Bln-1293H	3640	106	1974	[31]
5	I?	I?	1x?	grave A?	wood	Bln-1294	3585	106	1974	[31]
6	I?	I?	1x?	grave A?	humic acid from the sample Bln-1294	Bln-1294H	3620	106	1974	[31]
7	I?	I?	1x?	grave A?	wood	Bln-1295	3570	106	1974	[31]
8	I?	I?	1x?	grave A?	wood	Bln-1296	3645	106	1974	[31]
9	I?	I?	1x?	grave A?	humic acid from the sample Bln-1296	Bln-1296H	3710	79	1974	[31]
10	I?	I?	1x?	grave A?	wood	Bln-3218	3760	88	1985	[31]
11	IV	II	2	skeleton 2	human bone (metacarpus)	OxA-27967	3762	27	2015	[28]

After an anthropological re-analysis carried out in the new project, Burial 1 was found to contain the remains of a child aged 12–14 years, Burial 2 ‐ female, 25–35 years, and Burial 3 ‐ child, 9–10 years [32]. The remains from all three burials were then re-tested for aDNA in the Christian Albrecht University in Kiel. Burials 1 and 3 were confirmed to contain the remains of male children. In the case of Burial 2, the sex of the deceased was determined to be female. Additionally, the aDNA analyses, the results of which will be discussed in a systematic manner further, showed that all individuals deposited in examined burials were related to each other. Moreover, also aDNA of the male made under RISE shows his relationship to the rest of the deceased.

As discrepancies were found in the bio-archaeological assessment of Burial 2, further examination of the bones was undertaken. It confirmed that the vast majority of the remains belonged to a female, but a few may represent a male. After a detailed and multi-stage re-examination of the skeletal material, it can be concluded that the female skeleton was complete, while the male was represented by the only one bone examined in the RISE project.

Extended ^14^C AMS analyses were also carried out, resulting in four determinations for each burial (Table 4). Significantly, the four new dates of the Burial 2 are significantly younger than the OXA-27967 designation.

**Table 4:** Łęki Małe. List of radiocarbon dates used in the analyses reported in the text.

No.	Barrow	Sample data	Sample material	Lab. no.	Stratigraphy	BP	Standard deviation
1	I	Inv. 1953:210A p84_85	wood (oak)	Poz-115177	I-I1x	3675	30
2	I	Inv. 1953:210A p01	wood (oak)	Poz-115427	I-I1x	3710	30
3	I	Inv. 1953:210A p25	wood (oak)	Poz-115428	I-I1x	3735	30
4	I	Inv. 1953:210A p50	wood (oak)	Poz-115430	I-I1x	3620	30
5	I	Inv. 1953:210B p18-20	wood (oak)	Poz-116950	I-I1x	3645	35
6	I	Inv. 1953:210B p1-3	wood (oak)	Poz-116951	I-I1x	3640	40
7	I	Inv. 1953:210C p01	wood (oak)	Poz-116952	I-I1x	3710	30
8	I	Inv. 1953:210C p27-29	wood (oak)	Poz-117074	I-I1x	3740	35
9	I	Inv. 1953:210D p1-3	wood (oak)	Poz-117075	I-I1x	3700	35
10	I	Inv. 1953:210D p60-62	wood (oak)	Poz-117116	I-I1x	3690	35
11	I	Inv. 1953:210D p29-31	wood (oak)	Poz-117117	I-I1x	3645	35
12	II	Inv. 1933:1108	animal bone (undefined)	Poz-102120	II-VIy	2070	30
13	II	Inv.1933:1106/5r	human bone (rib)	Poz-112926	II-VIx	3525	35
14	II	Inv.1933:1106/5r	human bone (rib)	Poz-123309	II-VIx	3500	35
15	II	Inv.1933:1106/4r	human bone (rib)	Poz-112927	II-VIx	3460	35
16	II	Inv.1933:1106/4r	human bone (rib)	Poz-123310	II-VIx	3520	35
17	II	Inv.1933:1106/6	human bone (rib)	Poz-112928	II-VIx	3520	35
18	II	Inv.1933:1106/6	human bone (rib)	Poz-123311	II-VIx	3545	35
19	II	Inv.1933:1106/8s	human bone (part of the skull)	Poz-112929	II-VIx	3485	35
20	II	Inv.1933:1106/8s	human bone (part of the skull)	Poz-123312	II-VIx	3540	35
21	II	Inv.1933:1106/8r	human bone (rib)	Poz-112930	II-VIx	3670	35
22	II	Inv.1933:1106/8r, Cat.1933: 1106	human bone (rib)	Poz-123313	II-VIx	3620	35
23	II	Inv.1933:1106/13, Cat.1933: 1106	human bone (undefined)	Poz-112932	II-VIx	3565	35
24	II	Inv.1933:1106/13, Cat.1933: 1106	human bone (undefined)	Poz-123314	II-VIx	3630	35
25	II	Inv.1933:1106/12h1, Cat.1933: 1106	human bone (undefined)	Poz-112974	II-VIx	3485	30
26	II	Inv.1933:1106/12h1, Cat.1933: 1106	human bone (undefined)	Poz-123316	II-VIx	3505	35
27	II	Inv.1933:1106/3, Cat.1933: 1106	human bone (undefined)	Poz-112977	II-VIx	3445	30
28	II	Inv.1933:1106/3, Cat.1933: 1106	human bone (undefined)	Poz-123318	II-VIx	3500	30
29	II	Inv.1933:1106/1, Cat.1933: 1106	human bone (undefined)	Poz-112979	II-VIx	3560	30
30	II	Inv.1933:1106/1, Cat.1933: 1106	human bone (undefined)	Poz-123319	II-VIx	3515	30
31	II	Inv. 1106, IX.8, depth 3,27	animal bone (tibia, deer)	Poz-101067	II-VIy	1690	30
32	II	Inv. 1107, VII	animal bone (tibia, horse)	Poz-101068	II-VIx	3505	30
33	II	Inv. 1933:1106/2	human bone (undefined)	Poz-98591	II-VIx	3560	35
34	II	Inv. 1933:1106/4h	human bone (undefined)	Poz-98592	II-VIx	3535	35
35	II	Inv. 1933:1106/5h	human bone (undefined)	Poz-98593	II-VIx	3485	35
36	II	Inv. 1933:1106/7	human bone (undefined)	Poz-98594	II-VIy	1590	30
37	II	Inv. 1933:1106/8h	human bone (undefined)	Poz-98596	II-VIx	3570	35
38	II	Inv. 1933:1106/9	human bone (undefined)	Poz-98597	II-VIx	3585	35
39	II	Inv. 1933:1106/10	human bone (undefined)	Poz-98598	II-VIx	3515	35
40	II	Inv. 1933:1106/11	human bone (undefined)	Poz-98599	II-VIx	3515	30
41	II	Inv. 1933:1106/12h2	human bone (undefined)	Poz-98600	II-VIx	3535	35
42	II	Inv. 1106, IX.8, depth 3.27	animal bone (tibia, deer)	Poz-124549	II-VIy	1665	30
43	II	Inv. 1107, VII	animal bone (tibia, horse)	Poz-124550	II-VIx	3540	35
44	III	Inv. 1955:721a	animal bone (lower jaw, horse)	Poz-124551	III-V2ibis	3520	35
45	III	Inv. 1955:721a	animal bone (ulna, horse)	Poz-124552	III-V2iibis	3490	35
46	III	Inv. 1955:721a	animal bone (thoracic vertebra, horse)	Poz-124553	III-V2iiibis	3480	35
47	III	Inv. 1955:721a	animal bone (lumbar vertebra, horse)	Poz-124554	III-V2iiiibis	3520	35
48	III	Inv. 1955:721a	animal bones (undefined)	Poz-124557	III-V2iiiiibis	3520	35
49	III	Inv. 1955:721a	animal bone (lower jaw, dog)	Poz-124555	III-VII1ibis	135	30
50	III	Inv. 1955:701, grave A, pavement XIX	wood (oak)	Poz-97195	III-VI1i	4110	35
51	III	Inv. 1955:700, grave A, pavement XVIII	wood (oak)	Poz-97071	III-VI2i	3700	40
52	III	Inv. 1955:698d, grave A, pavement XII	wood (pine)	Poz-97073	III-VI3i	1595	35
53	III	Inv. 1955:696, grave A, pavement VI	wood (oak)	Poz-96960	III-VI4i	1395	30
54	III	Inv. 1955:697, grave A, pavement V	wood (pine)	Poz-97070	III-VI5i	1515	30
55	III	Inv. 1955:698, grave A, pavement IV	wood (pine)	Poz-96952	III-VI6i	2060	30
56	III	Inv. 1955:695a, grave A, pavement II	wood (oak)	Poz-96958	III-VI7i	1405	30
57	III	Inv. 1955:719, hearth 1	wood (birch)	Poz-96957	III-IV1i	3545	35
58	III	Inv. 1955:720, hearth 2	wood (pine)	Poz-97194	III-IV1ii	3510	35
59	III	Inv. 1955:715, grave B	wood (pine)	Poz-96961	III-V1i	3515	30
60	III	Inv. 1955:721a	animal bone (lower jaw, horse)	Poz-101069	III-V2i	3525	35
61	III	Inv. 1955:721a	animal bone (ulna, horse)	Poz-101070	III-V2ii	3535	35
62	III	Inv. 1955:721a	animal bone (thoracic vertebra, horse)	Poz-101071	III-V2iii	3520	35
63	III	Inv. 1955:721a	animal bone (lumbar vertebra, horse)	Poz-101073	III-V2iiii	3515	30
64	III	Inv. 1955:721a	animal bone (lower jaw, dog)	Poz-101074	III-VII1i	195	30
65	III	Inv. 1955:721a	animal bone (undefined)	Poz-101075	III-V2iiiii	3540	30
66	IV	Inv.1957:371, Cat.1957:1065, skeleton 1	human bone (undefined)	Poz-112973	IV-II1i	3620	30
67	IV	Inv.1957:371, Cat.1957:1065, skeleton 1	human bone (undefined)	Poz-122902	IV-II1ibis	3700	35
68	IV	Inv. 1957:1065, skeleton 1	human bone (undefined)	Poz-98601	IV-II1ii	3725	35
69	IV	Inv. 1957:1065, skeleton 1	human bone (undefined)	Poz-122903	IV-II1iibis	3745	50
70	IV	Inv. 1957:1068, skeleton 2	human bone (undefined)	Poz-98602	IV-II2i	3695	35
71	IV	Inv. 1957:1068, skeleton 2	human bone (undefined)	Poz-122904	IV-II2ibis	3680	30
72	IV	Inv.1957:371, Cat.1957:1068, skeleton 2	human bone (undefined)	Poz-112931	IV-II2ii	3670	35
73	IV	Inv.1957:371, Cat.1957:1068, skeleton 2	human bone (undefined)	Poz-122901	IV-II2iibis	3690	35
74	IV	skeleton 2	human bone (undefined)	OxA-27967	IV-II2iii	3762	27
75	IV	Inv.1957:371, Cat.1957:1069, skeleton 3	human bone (undefined)	Poz-112976	IV-II3i	3765	30
76	IV	Inv.1957:371, Cat.1957:1069, skeleton 3	human bone (undefined)	Poz-122900	IV-II3ibis	3685	35
77	IV	Inv. 1957:1069, skeleton 3	human bone (undefined)	Poz-98603	IV-II3ii	3630	35
78	IV	Inv. 1957:1069, skeleton 3	human bone (undefined)	Poz-122984	IV-II3iibis	3685	35
79	IV	Inv. 1957:170, next to the skeleton 3	animal bone (undefined)	Poz-98644	IV-II3iii	3715	35
80	IV	Inv. 1957:1070, next to the skeleton 3	animal bone (fragment of the skull, cattle)	Poz-101090	IV-II3iiii	3710	35
81	IV	Inv. 1957:1070, next to the skeleton 3	animal bone (fragment of the skull, cattle)	Poz-124560	IV-II3iiiibis	3670	35
82	IV	Inv. 1956:1899, hearth 1	wood (oak)	Poz-97067	IV-III1i	3760	40
83	IV	Inv. 1956:1899, hearth 1	wood (oak)	Poz-123249	IV-III1ibis	3790	30
84	IV	Inv. 1956:1900, hearth 2	wood (oak)	Poz-97069	IV-IV1i	3760	40
85	IV	Inv. 1956:1900, hearth 2	wood (oak)	Poz-123253	IV-IV1ibis	3810	40
86	IV	Inv. 1956:1903/-387, from the embankment	wood (oak)	Poz-96955	IV-V1i	3695	35
87	IV	Inv. 1956:1903/-387, from the embankment	wood (oak)	Poz-123252	IV-V1ibis	3720	35
88	IV	Inv. 1957:1066m, quarter II, depth 3.88	animal bone (metacarpal, cattle)	Poz-101089	IV-V2i	3560	35
89	IV	Inv. 1957:1066m, quarter II, depth 3.88	animal bone (metacarpal, cattle)	Poz-123962	IV-V2ibisbis	3460	35
90	IV	Inv. 1957:1066, quarter II, depth 3.88	animal bone (metacarpal, cattle)	Poz-124559	IV-V2ibis	3495	35
91	IV	Inv. 1956:1903/-332, from the embankment	wood (oak)	Poz-97074	IV-V3i	3720	35
92	IV	Inv. 1956:1903/-332, from the embankment	wood (oak)	Poz-123445	IV-V3ibis	3690	35
93	IV	Inv. 1956:1903/-84, from the embankment	wood (willow)	Poz-96956	IV-V4i	3730	35
94	IV	Inv. 1956:1903/-84, from the embankment	wood (willow)	Poz-123251	IV-V4ibis	3715	30
95	IV	Inv. 1956:1903, from the embankment	wood (oak)	Poz-97068	IV-V5i	3780	40
96	IV	Inv. 1956:1903, from the embankment	wood (oak)	Poz-123250	IV-V5ibis	3780	30
97	IV	Inv. 1956:1903/-390, from the embankment	wood (pine)	Poz-96954	IV-VI1i	3505	35
98	IV	Inv. 1956:1903/-390, from the embankment	wood (pine)	Poz-123254	IV-VI1ibis	3445	35
99	IV	Inv. 1956:350, central grave, depth 3.16, from NS 1.9, from EW 4.4	wood (diffuse-porous hardwood)	Poz-97175	IV-VI2i	3545	30
100	IV	Inv. 1957:1066r, quarter I, depth 3.91	animal bone (rib)	Poz-101076	IV-VII1i	265	30
101	IV	Inv. 1957:1066bis, quarter I, depth 3.91	animal bone (rib)	Poz-121183	IV-VII1ibis	250	30
102	IV	Inv. 1957:1066, quarter I, depth 3.91	animal bone (rib)	Poz-124558	IV-VII1ibisbis	230	30

Thus, the inconsistency of the bio-archaeological results of Burial 2 is accompanied by the inconsistency of the chronometric results. They are so significant that no simple explanation can be found for them, e.g. as a consequence of faulty exploration/storage resulting in mixed remains from different burials or mistakes in radiocarbon and archaeogenetic laboratories. The discussed inconsistencies seem to have revealed a fact that was not observed during the exploration and first anthropological studies: the deposition in Burial 2 of the remains of two individuals.

For this reason, it is appropriate to separate two individuals in Burial 2:

- Individual 2a - a female whose body was probably intact in the burial; examined in our project and dated with four ^14^C determinations Poz-98602 3695±35 BP; Poz-122904 3680±30 BP; Poz-112931 3670±35 BP; Poz-122901 3690±35 BP (Table 4);
- Individual 2b –a male whose only selected bones (from which only one has preserved to date) were deposited with the body of a female; one preserved bone was examined in the RISE project (as sample RISE431) and dated with one ^14^C determination OxA-27967 3762±27 BP (Table 3).

The outlined problem has important implications for the absolute chronology of the Barrow IV, as Burials 1, 2 and 3 belong to the oldest stratigraphic level (II) in the barrow. Thus, establishing their absolute chronology affects all further considerations. In particular, it must be resolved whether the dating of Individual 2b is crucial in determining the time of deposition of Burial 2 as well as nearby Burials 1 and 3. Following the above presented conclusion, we believe that the OxA-27967 determination does not relate to the chronology of either Burial 2 or two other burials located nearby, but probably belongs to human bones from an older male individual, which accompanied as a secondary deposition the female in Burial 2. Consequently the OxA-27967 determination does not date an activity at Barrow IV.

Thus, the age of the three burials will be determined on the basis of 12 convergent datings from the Poznań Radiocarbon Laboratory (Table 4). The OxA determination will be used only in a limited way.

### Absolute chronology: Results of radiocarbon and dendrochronological analyses

Before our project started, ten conventional and one AMS ^14^C dates were available for the Łęki Małe cemetery (Table 3). Although some of the conventional dates were measured quite some time ago, all radiocarbon determinations correspond with a period in the late 3rd and the early 2nd millennium BC.

In the new approach altogether 70 samples were submitted to the Poznań Radiocarbon Laboratory [26], of which three bone samples did not retain collagen and could not be utilized for measurement. Successful ^14^C AMS analyses were therefore carried out on 67 samples: 66 samples derived from Barrows I-IV and one sample was measured from around Barrow IV (Poz-96953; 2485±35 BP). The last sample is not considered in this paper. In total, 101 ^14^C determinations were obtained from the 66 samples, with 31 samples yielding two determinations each and two samples producing three dates each (Table 4). Including the date OxA-27967, the following number of ^14^C determinations was made for each burial mound: Barrow I–n = 11, Barrow II–n = 32, Barrow III–n = 22 and Barrow IV–n = 37. In total, this article uses 102 radiocarbon determinations. The stable isotopes values (^13^C and ^15^N) of the samples demonstrate the lack of the reservoir effects (Table 5) and we were able to link each sample to one of the seven stratigraphic levels listed above.

**Table 5:** Łęki Małe. Determinations of stable isotopes in human bones.

No.	Barrow	Lab. no.	^14^C - BP	Standard deviation	Stable isotopes (^13^C, ^15^N)
1	II	Poz-112926	3510	35	δ^13^C = -20.9‰, δ^15^N = 11.1‰
2	II	Poz-112927	3435	35	δ^13^C = -21‰, δ^15^N = 11.1‰
3	II	Poz-112928	3490	35	δ^13^C = -21‰, δ^15^N = 11‰
4	II	Poz-112929	3455	35	δ^13^C = -20.9‰, δ^15^N = 11.5‰
5	II	Poz-112930	3635	35	δ^13^C = -20.9‰, δ^15^N 10.8‰
6	II	Poz-112932	3530	35	δ^13^C = -20.4‰, δ^15^N = 12.3‰
7	II	Poz-112974	3485	30	δ^13^C = -20.5‰, δ^15^N = 12.1‰
8	II	Poz-112977	3445	30	δ^13^C = -20.7‰, δ^15^N = 12‰
9	II	Poz-112979	3560	30	δ^13^C = -21.1‰, δ^15^N = 11.2‰
10	II	Poz-98591	3560	35	δ^13^C = -21.1‰, δ^15^N = 11.1‰
11	II	Poz-98592	3535	35	δ^13^C = -21‰, δ^15^N = 11.1‰
12	II	Poz-98593	3485	35	δ^13^C = -20.9‰, δ^15^N = 11.3‰
13	II	Poz-98594	1590	30	δ^13^C = -21‰, δ^15^N = 5.4‰
14	II	Poz-98596	3570	35	δ^13^C = -21‰, δ^15^N = 11.2‰
15	II	Poz-98597	3585	35	δ^13^C = -20.9‰, δ^15^N = 11.5‰
16	II	Poz-98598	3515	35	δ^13^C = -21.1‰, δ^15^N = 11.1‰
17	II	Poz-98599	3515	30	δ^13^C = -21‰, δ^15^N = 11‰
18	II	Poz-98600	3535	35	δ^13^C = -20.5‰, δ^15^N = 12.1‰
19	IV	Poz-112973	3620	30	δ^13^C = -21.1‰, δ^15^N = 10.6‰
20	IV	Poz-98601	3725	35	δ^13^C = -21.2‰, δ^15^N = 10.7‰
21	IV	Poz-98602	3695	35	δ^13^C = -20.5‰, δ^15^N = 12.1‰
22	IV	Poz-112931	3670	35	δ^13^C = -20.9‰, δ^15^N = 10.4‰
23	IV	Poz-112976	3765	30	δ^13^C = -21.2‰, δ^15^N = 10.2‰
24	IV	Poz-98603	3630	35	δ^13^C = -21.1‰, δ^15^N = 10.5 ‰

Exclusively in the case of Barrow I, the preservation of charred wood from the central tomb allowed dendrochronological analysis and wiggle matching of the ^14^C determinations.

In the following section, we will present the results for each barrow individually, beginning with Barrow IV at the south-eastern end of the cemetery and preceding in order to the north-west.

### Barrow IV

We measured 19 organic samples from Barrow IV, resulting in 37 radiocarbon determinations (Table 6). The samples were taken from human (n = 7) and animal (n = 4) bones, and charcoal (n = 8). Three of the samples have one ^14^C determination; 14 samples have two (double), and in the case of two, three determinations were obtained. Based on the known, precise stratigraphic position of each sample, we established a Harris matrix (Fig 6A).

**Table 6:** Łęki Małe, Barrow IV. Results of ^14^C dating.

Stratigraphic Level	Context	Sample	Unit	Material	Years / increments	Lab. No. Poz-	^14^C BP	1-sigma	%coll/C/N
II	1	1957:1065/1	burial 1	human bone	14–16	112973	3620	30	5.1%/3.26
						122902	3700	35	
		1957:1065/2	burial 1	human bone	14–16	98601	3725	35	3.3%/3.25
						122903	3745	50	
						**Average**	**3684**	**18**	
	2	1957:1068a/1	burial 2a	human bone	30–40	98602	3695	35	5.9%/3.20
						122904	3680	30	
		1957:1068a/2	burial 2a	human bone	30–40	112931	3670	35	6.0%/3.25
						122901	3690	35	
						**Average**	**3682**	**17**	
		1957:1068b	burial 2b	human bones		OxA-27967	3762	27	
	3	1957:1069/1	burial 3	human bone	9–10	112976	3765	30	4.1%/3.24
						122900	3685	35	
		1957:1069/2	burial 3	human bone	9–10	98603	3630	35	11.0%/3.21
						122984	3685	35	
						**Average**	**3698**	**17**	
		1957:170	next to burial 3	animal bone	‐‐	98644	**3715**	**35**	10.0%/3.21
		1957:1070	next to burial 3	cattle skull	‐‐	101090	3710	35	4.9%/3.23
						124560	3670	35	
						**Average**	**3690**	**25**	
III	1	1956:1899	hearth I	oak	50?	97067	3760	40	
						123249	3790	30	
						**Average**	**3779**	**25**	
IV	1	1956:1900	hearth 2	oak	50?	97069	3760	40	
						123253	3810	40	
						**Average**	**3785**	**29**	
V	1	1956:1903/-387	from the embankment	oak	300?	96955	3695	35	
						123252	3720	35	
						**Average**	**3708**	**25**	
	2	1957:1066m	quarter II, depth 3.88	cattle metacarpal	‐‐	101089	3560	35	10.5%/3.22
						123962	3460	35	
						124559	3495	35	
						**Average**	**3505**	**21**	
	3	1956:1903/-332	from the embankment	oak	300?	97074	3720	35	
						123445	3690	35	
						**Average**	**3705**	**25**	
	4	1956:1903/-84	from the embankment	willow	30?	96956	3730	35	
						123251	3715	30	
						**Average**	**3721**	**23**	
	5	1956:1903	from the embankment	oak	300?	97068	3780	40	
						123250	3780	30	
						**Average**	**3780**	**24**	
VI	1	1956:1903/-390	from the embankment	pine	30?	96954	3505	35	
						123254	3445	35	
						**Average**	**3475**	**25**	
	2	1956:350	central grave, depth 3.16	diffuse-porous hardwood	30?	97175	**3545**	**30**	
VII	1	1957:1066r	quarter I, depth 3.91	animal rib	‐‐	101076	265	30	10.7%/3.19
						121183	250	30	
						124558	230	30	
						**Average**	**248**	**18**	

The stratigraphically earliest samples belong to human individuals 1, 2a, 2b, and 3 (stratigraphic level II, Table 6); radiocarbon determinations on these samples also represent the oldest dates, with the oldest one obtained for Individual 2b (OxA-27967 3762±27 BP), who, however, probably died some time before part of his remains were placed in Burial 2. Nevertheless, dates from two hearths documented directly above burials (in the stratigraphic levels III and IV) confirm the early dating of these graves, as they “seal” the oldest human activities at the barrow: the burial of individuals.

Additional dates represent successive phases of human activity taking place in the mound in the Early Bronze Age (stratigraphic levels V and VI). The stratigraphically latest sample, from the intrusion pit (stratigraphic level VII) destroying the central tomb, also returned the youngest date (Poz-101076 265±30 BP; Poz-121183 250±30 BP; Poz-124558 230±30 BP), supporting our stratigraphic model. This action may also mark the time of the destruction and pillage of Barrow IV.

### Barrow III

We measured 16 organic samples from Barrow III, producing 22 radiocarbon determinations (Table 7). Six of the samples have two determinations. The samples include charcoal (n = 10) and animal bones (n = 6).

**Table 7:** Łęki Małe, Barrow III. Results of ^14^C dating.

Stratigraphic Level	Context	Sample	Unit	Material	Years/ increments	Lab. No. Poz-	14C BP	1-sigma	%coll/C/N
IV	1	1955:719	hearth 1	birch	30	96957	3545	35	
		1955:720	hearth 2	pine	30	97194	3510	35	
V	1	1955:715	grave B	pine	30	96961	3515	30	
	2	1955:721a-1		horse, lower jaw	‐‐	101069	3525	35	5.7%/3.20
						124551	3520	35	
						**Average**	**3523**	**25**	
		1955:721a-2		horse, ulna	‐‐	101070	3535	35	7.7%/3.22
						124552	3490	35	
						**Average**	**3513**	**25**	
		1955:721a-3		horse, thoracic vertebra	‐‐	101071	3520	35	7.4%/3.21
						124553	3480	35	
						**Average**	**3500**	**25**	
		1955:721a-4		horse, lumbar vertebra	‐‐	101073	3515	30	9.0%/3.22
						124554	3520	35	
						**Average**	**3517**	**23**	
		1955:721a-6		animal bones, undefined	‐‐	101075	3540	30	4.7%/3.23
						124557	3520	35	
						**Average**	**3532**	**23**	
VI	1	1955:701	grave A, pavement XIX	oak	300?	97195	4110	35	
	2	1955:700	grave A, pavement XVIII	oak	300?	97071	3700	40	
	3	1955:698d	grave A, pavement XII	pine	50?	97073	1595	35	
	4	1955:696	grave A, pavement VI	oak	30?	96960	1395	30	
	5	1955:697	grave A, pavement V	pine	30?	97070	1515	30	
	6	1955:698	grave A, pavement IV	pine	30?	96952	2060	30	
	7	1955:695a	grave A, pavement II	oak	30?	96958	1405	30	
VII	1	1955:721a-5	1955:721a	dog, lower jaw	‐‐	101074	195	30	7.4%/3.24
						124555	135	30	
						**Average**	**165**	**22**	

While we recognize the possibility of an old-wood effect (e.g. [33]) for radiocarbon determinations made on charred wood samples, only charcoal has survived from one of the most important contexts of Barrow III. Stone “pavements”, located one below the other (each made up of multiple stones) and found inside the central Tomb A, which were documented during excavation in the 1950s, likely mark the destroyed original stone construction of the central tomb. Unfortunately, no preserved human or animal bones were found in this context [34,35]. We therefore included seven charcoal samples collected from seven “pavements” in the radiocarbon measurement (Fig 6B), but identified the species of wood and made adjustments based on the lifespan of these species and the principles described below.

Four of the samples of charcoal were oak (*Quercus L*.) and three were pine (*Pinus L*.). As a rule, the ^14^C determinations for these samples follow the stratigraphic order of the “pavements”. However, based on the ^14^C determinations, the majority of samples are unlikely to have been related to the construction of the central Tomb A. Only the sample taken from “pavement” XVIII may represent material remaining from the central Tomb A. The earliest date (”pavement” XIX; Poz-97195 4110±35) seems to represent re-deposited Neolithic material (perhaps from stage III; see Table 2). The rest of the dates from “pavements” tend to be much younger, falling in the first centuries AD. They thus represent the re- use of this barrow in later times, and the youngest date may indicate the time when the central Tomb A was destroyed.

Several other features distinguished during the excavations, namely Hearth no. 1, Hearth no. 2, and Grave B, were also dated using charcoal samples: birch (*Betula L*.; n = 1) and pine (*Pinus L*., n = 2). We argue that wood burnt in both hearths was unlikely to have been from trunks of trees that grew for many years, allowing us to exclude the old-wood effect. The two hearths belong to stratigraphic level IV, the surface of the primary humus. Grave B was embedded in the mound (stratigraphic level V), and its bottom touched (but did not cut) the surface of the primary humus. The features revealed in stratigraphic levels IV and V form a micro-sequence, with the hearths representing the stage of ritual activities that could have been held while the barrow was being constructed.

Six (n = 6) animal bones were found in the mound (stratigraphic level V) (Table 7). One *Canis familiaris* (dog) bone returned a modern date, while the other five bones date to the Early Bronze Age.

### Barrow II

The excavated area covered only the central portion of the barrow, likely including the central tomb, as indicated by partly preserved rounded remnants of a stone structure in the northern part of the unit marking the tomb’s extent. The excavation in question reveals, above all, the extent of damage to the central tomb (stratigraphic level VI, Fig 6C). A total of 21 samples were analyzed: 18 of human bone and three of animal bone. 11 of the samples produced two ^14^C determinations. Thus, there are a total of 32 determinations, of which solely three dates point to a period other than the Early Bronze Age (Table 8).

**Table 8:** Łęki Małe, Barrow II. Results of ^14^C dating.

Stratigraphic Level	Context	Sample	Unit	Material	Lab. No. Poz-	14C BP	1-sigma	%coll/C/N
VI	X	Inv.1933:1106/1		human bone	112979	3560	30	6.5%/3.20
					123319	3515	30	
					Average	3538	22	
		1933:1106/2		human bone	98591	3560	35	6.7%/3.19
		Inv.1933:1106/3		human bone	112977	3445	30	5.9%/3.21
					123318	3500	30	
					Average	3473	22	
		Inv.1933:1106/4r		human rib	112927	3460	35	10.4%/3.22
					123310	3520	35	
					Average	3490	25	
		1933:1106/4h		human bone	98592	3535	35	6.0%/3.21
		Inv.1933:1106/5r		human rib	112926	3525	35	4.8%/3.20
					123309	3500	35	
					Average	3513	25	
		1933:1106/5h		human bone	98593	3485	35	7.2%/3.19
		Inv.1933:1106/6		human rib	112928	3520	35	6.3%/3.23
					123311	3545	35	
					Average	3533	25	
		Inv.1933:1106/8s		human skull	112929	3485	35	6.5%/3.20
					123312	3540	35	
					Average	3513	25	
		Inv.1933:1106/8r		human rib	112930	3670	35	1.8%/3.20
					123313	3620	35	
					Average	3645	25	
		1933:1106/8h		human bone	98596	3570	35	7.2%/3.22
		1933:1106/9		bone	98597	3585	35	5.7%/3.22
		1933:1106/10		bone	98598	3515	35	12.5%/3.23
		1933:1106/11		bone	98599	3515	30	3.6%/3.14
		Inv.1933:1106/12h1		human bone	112974	3485	30	4.9%/3.20
					123316	3505	35	
					Average	3493	23	
		1933:1106/12h2		bone	98600	3535	35	4.7%/3.17
		Inv.1933:1106/13		human bone	112932	3565	35	5.8%/3.21
					123314	3630	35	
					Average	3598	25	
		Inv. 1107	VII	horse, tibia	101068	3505	30	5.1%/3.24
					124550	3540	35	
					Average	3520	23	
	Y	Inv. 1106	IX.8, depth 3.27	deer, tibia	101067	1690	30	6.3%/3.25
					124549	1665	30	
					Average	1678	22	
		1933:1108		bone	102120	2070	30	5.2%/3.27
		1933:1106/7		bone	98594	1590	30	6.7%/3.23

The human remains derive from the area of the destroyed central tomb. However, they are unlikely to include the remains of the person for whom the tomb was raised, although this individual may be included in the second group discussed below. The human remains can be divided into two categories: individuals preserved as a group represented by dozens of bones (probably *in situ*) and individuals that have been identified by individual bones (possibly re-deposited). Anthropological analysis revealed that the first of these groups includes bones of children and young individuals: two Infans I (3–4 and 6–7 years old), two Infans II (7–8 years old) and a female 20–25 years old. The individuals identified through individual bones are all adults, though their sex and age at death unfortunately could not be determined. We argue that the remains belonging to both of these groups may represent people who were buried immediately following the interment of the central individual, during the initial construction of the barrow.

However, a robbery distorted stratigraphic observations and some bones with Early Bronze Age dates could also document use of Barrow II after its construction by the Únětice community (stratigraphic level V?). This concern mainly the ^14^C dates from single bones which were most likely moved as a result of post-depositional processes.

### Barrow I

All radiocarbon determinations for Barrow I (Table 9) were measured on wood samples, and were meant to assist the dendrochronological analysis. Unfortunately, the dendrochronology did not provide absolute dating. The inner part of the central tomb of Barrow I was made of oak (*Quercus L*.) (Fig 6D). Unlike in the case of the hearths from Barrow III, oak used for construction could have been selected from older trees with larger trunks. Dendrological observations did not reveal any bark or sapwood which allows us to suppose that the entire series of dates for Barrow I is loaded with the old wood effect. Likewise, the context of the ^14^C determination performed earlier in Michigan and Groningen laboratories (Table 3) cannot be determined.

**Table 9:** Łęki Małe, Barrow I. Results of ^14^C dating.

Stratigraphic Level	Context	Sample	Material	Years/ increments	Lab. No. Poz-	^14^C BP	1-sigma
I	1x	1953:210A_p01	oak		115427	3710	30
				24			
		1953:210A_p25	oak		115428	3735	30
				25			
		1953:210A_p50	oak		115430	3620	30
				85			
		1953:210A_p84_85	oak		115177	3675	30
				< = 215			
		1953:210B p1-3	oak		116951	3640	40
				17			
		1953:210B p18-20	oak		116950	3645	35
				< = 280			
		1953:210C p01	oak		116952	3710	30
				27			
		1953:210C p27-29	oak		117074	3740	35
				< = 260			
		1953:210D p1-3	oak		117075	3700	35
				28			
		1953:210D p29-31	oak		117117	3645	35
				31			
		1953:210D p60-62	oak		117116	3690	35
				33			
				< = 205			

## Integrating stratigraphy, radiocarbon and dendrochronology of barrows

We subjected the radiocarbon determinations and dendrochronological results to a detailed analysis of the mounds’ stratigraphy.

### Barrow IV

Table 6 presents our proposed improvements to the dating of samples based on considerations of the age at which an individual died and the limited rate of carbon exchange in bones [36] for the case of human bones and the age of the sampled trees in the case of charcoal. We argue that oak (*Quercus L*.) charcoals from hearths likely represent newly growing trees less than 50 years old. However, oak charcoals not clearly from hearth contexts may represent the remnants of timber constructions; these trees may have been up to 300 years old at the time of felling. We estimate other wood species (willow, pine) to have a short lifespan (up to 30 years).

**Fig 7:**
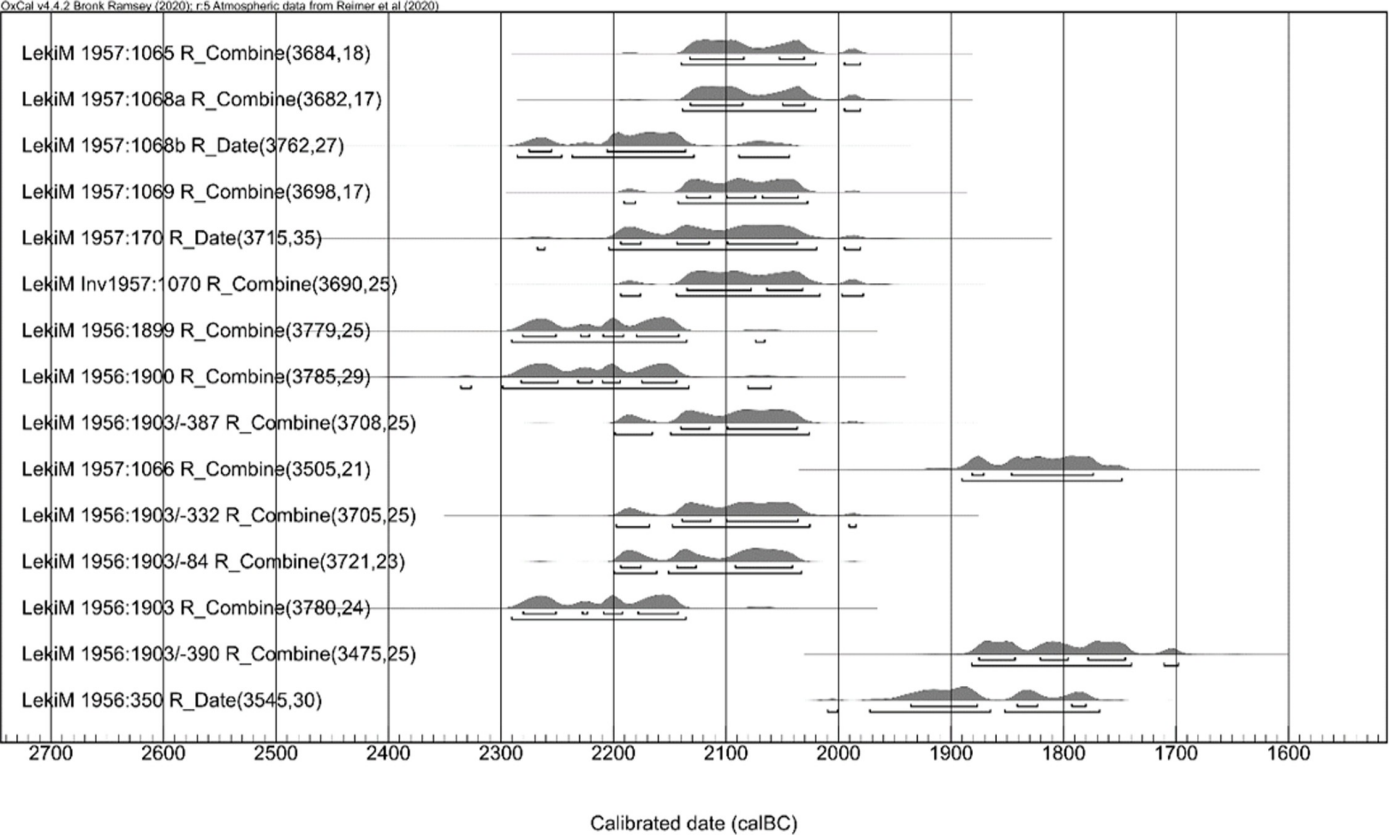
Łęki Małe, Barrow IV. The results of the calibration of radiocarbon dates. R_Combine is the calibration of the mean (calculated by Oxcal) over the ^14^C ages obtained for the same wood sample or for bone samples from the same skeleton. Modern dates from stratigraphic level VII are not included.

As the first step in recalculating the data, we took the average of dates received from samples of the same material (Table 6) providing, in turn, the basis for calibration (Fig 7). R_Combine stands for calibration of the mean ^14^C ages measured for the same sample (wood or human or animal bones from the same individual). The calibration was computed with the aid of the program OxCal v. 4.2.3 [37,38].

**Fig 8:**
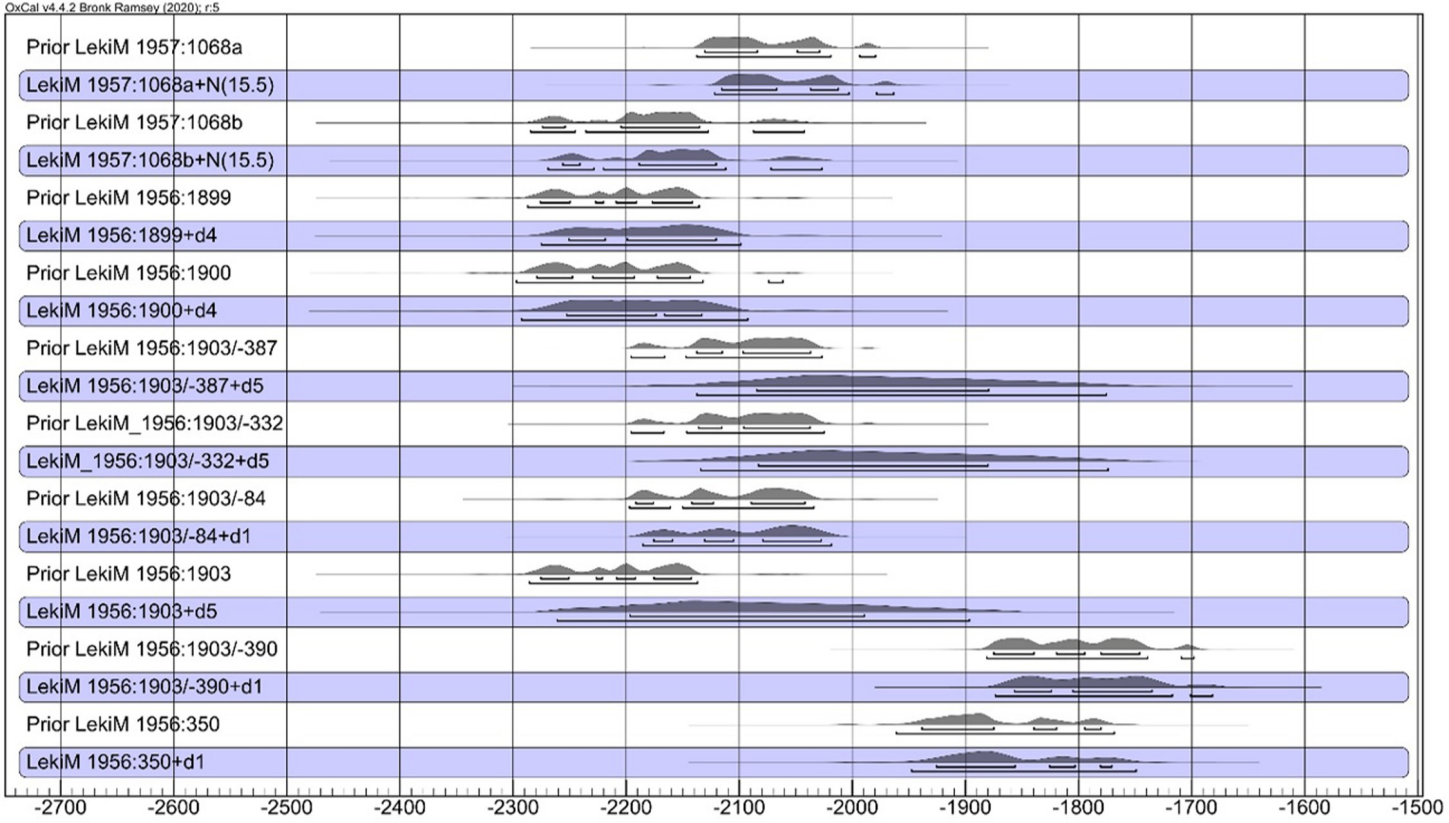
Łęki Małe, Barrow IV. Probability distributions of the dates of the wood samples and (against a blue background) the dates of felling (or death of the individual) from which these samples were derived. The time interval between the formation of the dated wood sample and the felling of the tree was assumed to be exponential and to be in the range of 0–30 years (correction "d1"), 0–50 years (“d4”) or 0–300 years (“d5”). For a bone sample (ŁękiM 1957: 1068) of an individual who died at the age of 30–40 years, the rejuvenation of the calendar date was applied with a value derived from the normal distribution N (15.5).

We then made adjustments to the lifespan of organic material in samples, as discussed above (cf. Table 4). For wood samples, we applied an approach analogous to that proposed by Makarowicz et al. [39]. Fig 8 illustrates the adjustments, showing the probability distributions of dates of wood and bone samples, and the dates of tree felling or death of the individuals (as the blue background).

**Fig 9:**
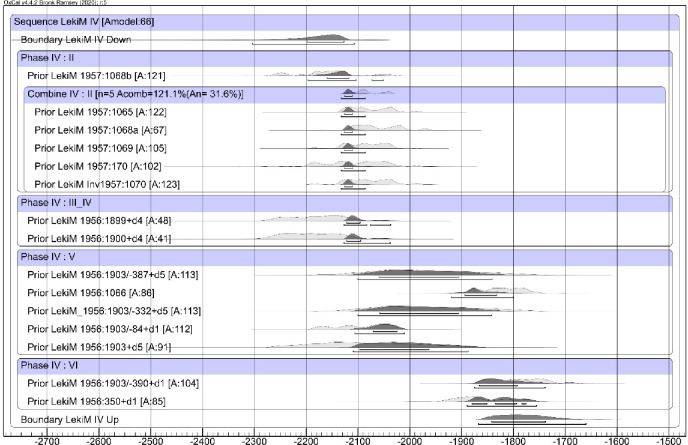
Łęki Małe, Barrow IV. Bayesian date set modeling results. The dates of samples from stratigraphic levels II, III-IV, V and VI were assumed to form an interrupted time sequence. All samples from stratigraphic level II were assumed to represent the same calendar date.

Subsequently, we combined the modified dates with our stratigraphic model using Bayesian modeling (Fig 9). The recalculated dates suggest that Barrow IV was formed during the Early Bronze Age, with three phases defined in absolute chronological terms: the instant of barrow’s creation ‐ 2130–2120 BC, the period of its use in the Early Bronze Age ‐ from the early 21st to the early 19th century, and the moment of the central tomb’s destruction ‐ 1800/1775 BC.

**Fig 10:**
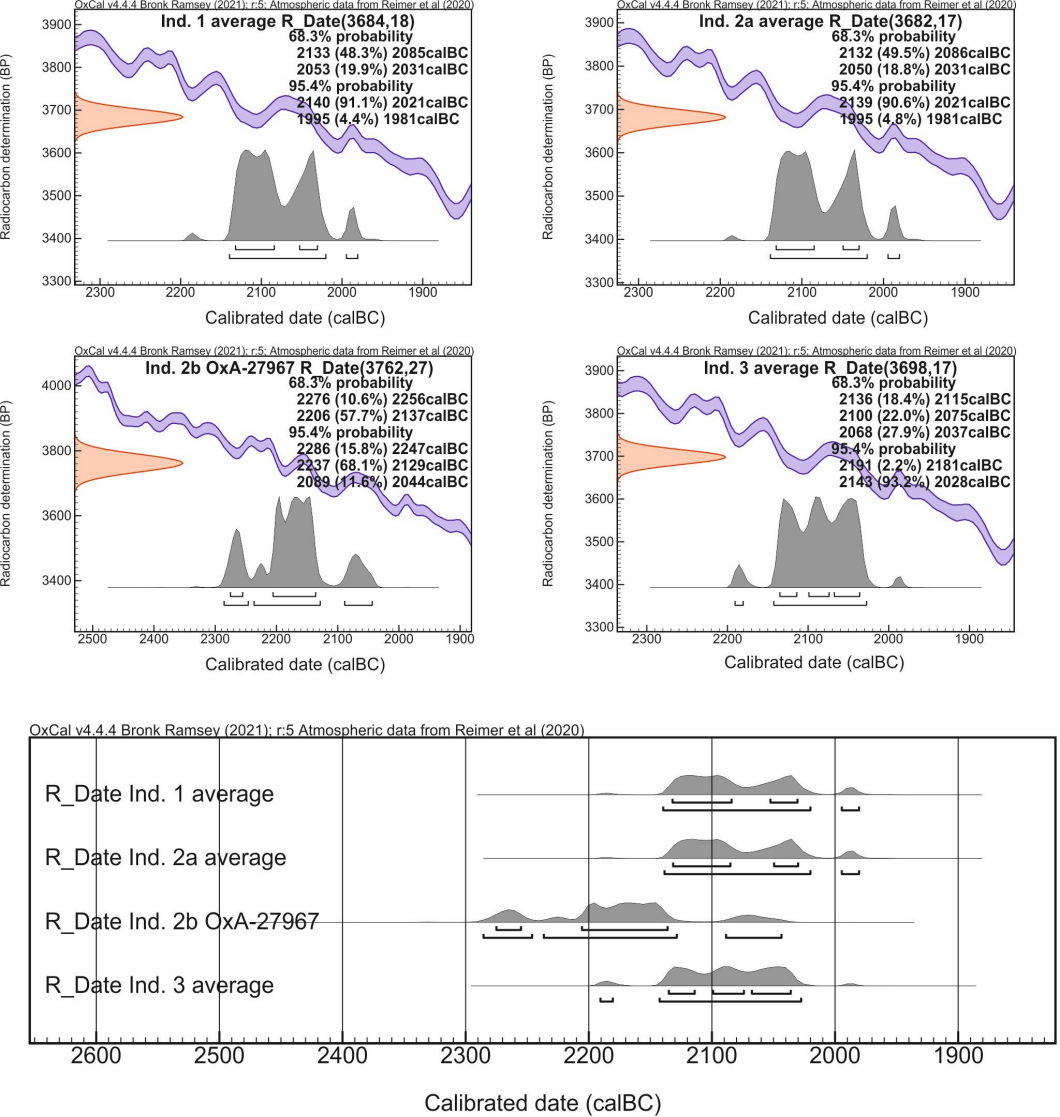
Łęki Małe, Barrow IV. Differentiation of radiocarbon chronology of Individuals: 1, 2a, 2b and 3.

Phase 1 is based on the dates for the interred individuals. The dating for Individual 2b (OxA-27967) was slightly older than the determinations for Individuals 1, 2a and 3 (Table 6 and Fig 10). According to above considerations (see above: section on the special case of bio-archaeological results from barrow IV) Individual 2b was a male who died earlier and may have been one generation older than the rest of deceased buried in side graves in Barrow IV (Individuals 1, 2a and 3). The date of death of Individual 2b is the earliest in the cemetery, however, it must be considered that his burial could have taken place also at a later date, alongside those of Individuals 1, 2a and 3 (single bones attached to burial 2a?). In this case, it could be assumed, based on stratigraphy, that the graves have been created as a result of a one-time action involving the erection of the barrow dated to ca. 2130–2120 BC. Furthermore, this assumption is the basis of the R_Combine function applied to calculate the dates of level II (Fig 9). If these dates are instead grouped using the Phase function, implying a more or less equal distribution of dates over a finite period, the model becomes no longer internally consistent with an A_model_ compliance index significantly lower than 60 [40].

Hearth no. 1 (stratigraphic level III) and 2 (stratigraphic level IV), are both slightly younger, but still remain within the late 22nd century BC. Including an assumption that the date of the sample from level III is significantly older than this from level IV caused model inconsistencies, a problem solved only by allowing the dates of both levels to be identical. The ^14^C determinations, therefore, demonstrate that the first stages of formation of the barrow associated with levels II, and then III and IV developed in a short time, here referred to as Phase 1.

Stratigraphic level V marks the next Phase 2 where the barrow was raised and used during the Early Bronze Age. While five ^14^C determinations are available for this level, all produce diverging results. Nevertheless, they fall to the Early Bronze Age, from the early 21st until the beginning of the 19th century BC. Around 1800/1775 BC a deep ditch was cut into the center of the mound, destroying the main tomb and marking the last phase when Early Bronze Age communities used Barrow IV.

### Barrow III

**Fig 11:**
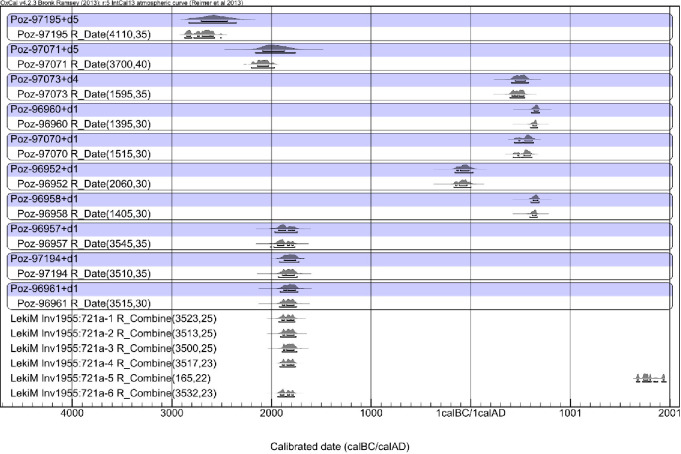
Łęki Małe, Barrow III. Probability distributions of the dates of the wood samples and (against a blue background) the dates of harvesting the trees from which the samples were derived. The time interval between the formation of the dated wood sample and the felling of the tree was assumed to be exponential and to be in the range of 0–30 years (correction "d1"), 0–50 years (“d4”) or 0–300 years (“d5”).

**Fig 12:**
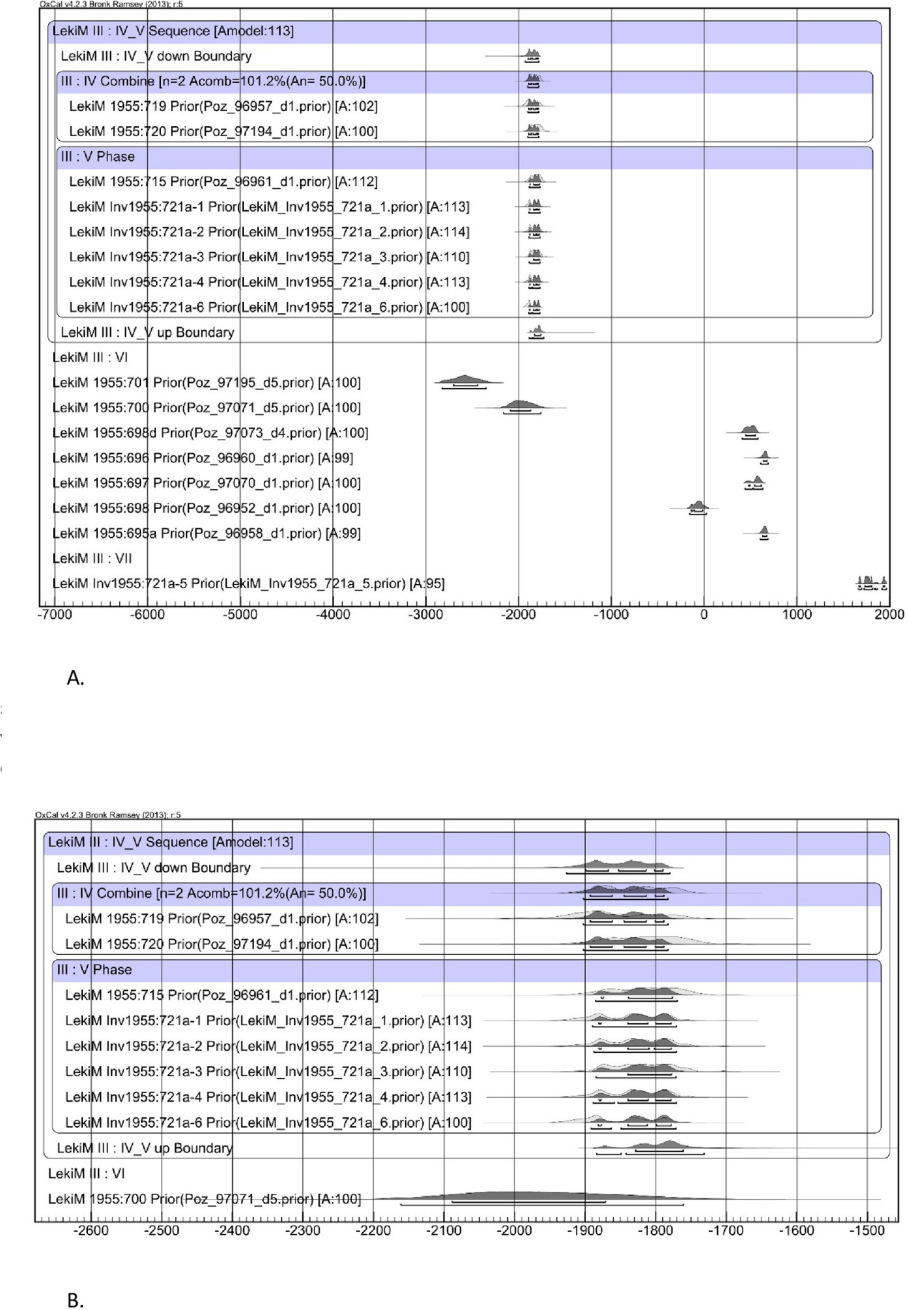
Łęki Małe, Barrow III. **A. Bayesian date set modeling results**. It was assumed that the sampling dates for stratigraphic level IV are identical, and that the dates for samples from stratigraphic level V that are younger than these fall within one phase. The sampling dates for stratigraphic levels VI and VII were calibrated independently. **B. Barrow III, as in Fig 12.A, with a horizontal scale covering only the stratigraphic levels IV and V date range**.

The first procedure in recalculating the data was to average the duplicate dates (Table 7). Then, the probability distribution (Fig 11) was generated in two versions: without correction and with correction of the wood lifespan for a given sample (the last one is seen against the blue background in Fig 11). In Bayesian modeling, only the latter of the versions mentioned above have been used (Fig 12A and 12B).

The oldest dated stratigraphic features are hearths 1 and 2 (stratigraphic level IV), forming a micro-sequence with Grave B (level V). This group is generally dated to the 19th century BC (Table 7). In turn, the construction time of the central tomb is indirectly indicated by a ^14^C determination from “pavement” XVIII, that is around 2000 BC (Table 7). All other determinations from level V probably come from a single horse (*Equus caballus*) individual and can also be placed in the 19th century BC (Table 7). Radiocarbon determinations for the younger “pavements” (XII, VI, V, IV and II) demonstrate a considerable intermingling and their distribution reflects the in-filling of the intrusion pit that destroyed the center of the barrow. The youngest ^14^C determination, from sample Poz-96958 1400±30 BP, provides a *terminus ante quem* for the digging of this pit ca. 600/650 AD.

Thus, we can provide dates for three stages in the use of Barrow III. The construction of this burial mound may have taken place as early as 2000 BC, as indicated by oak wood from the “pavement” XVIII we assume comes from the wood used to build the central tomb. A much firmer basis for dating of this event is provided by ^14^C determinations from the two hearths we consider remnants of ritual activity associated with the barrow’s creation. They indicate the 19th century BC for these activities. This slightly later chronology is also supported by dates for the stratigraphically younger level V (the mound) which indicate the mound was constructed in the 19th century BC. Finally, the most evident signs of Barrow III’s destruction can be attributed to ca. 600–650 AD.

### Barrow II

As the stratigraphic data from Barrow II is very scarce, and only its central part was excavated, we assume that the ^14^C determinations are dating the building and use of the central tomb (Table 8).

**Fig 13:**
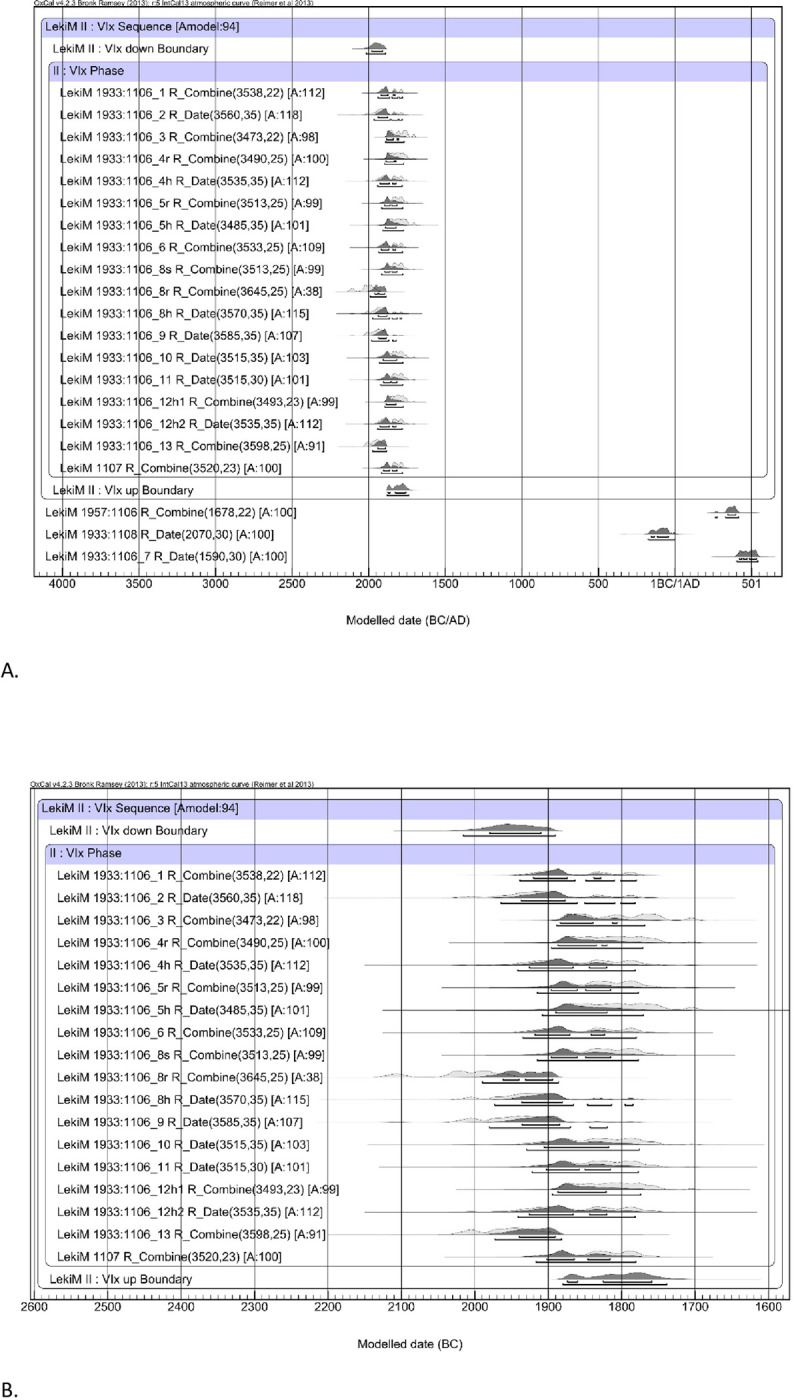
Łęki Małe, Barrow II. **A. Bayesian date set modeling results**. VIx stratigraphic level sample dates are assumed to be within one phase. The dates of the samples from stratigraphic level VIy were calibrated as independent. **B. Barrow II, as in Fig 13.A, with a horizontal scale covering only the VIx stratigraphic level date range**.

Following a procedure identical to the previous cases, Bayesian modeling results were obtained (Fig 13A and 13B). They suggest that the building and use of this tomb fall between the 20th and late 19th/early 18th century BC.

### Barrow I

**Fig 14:**
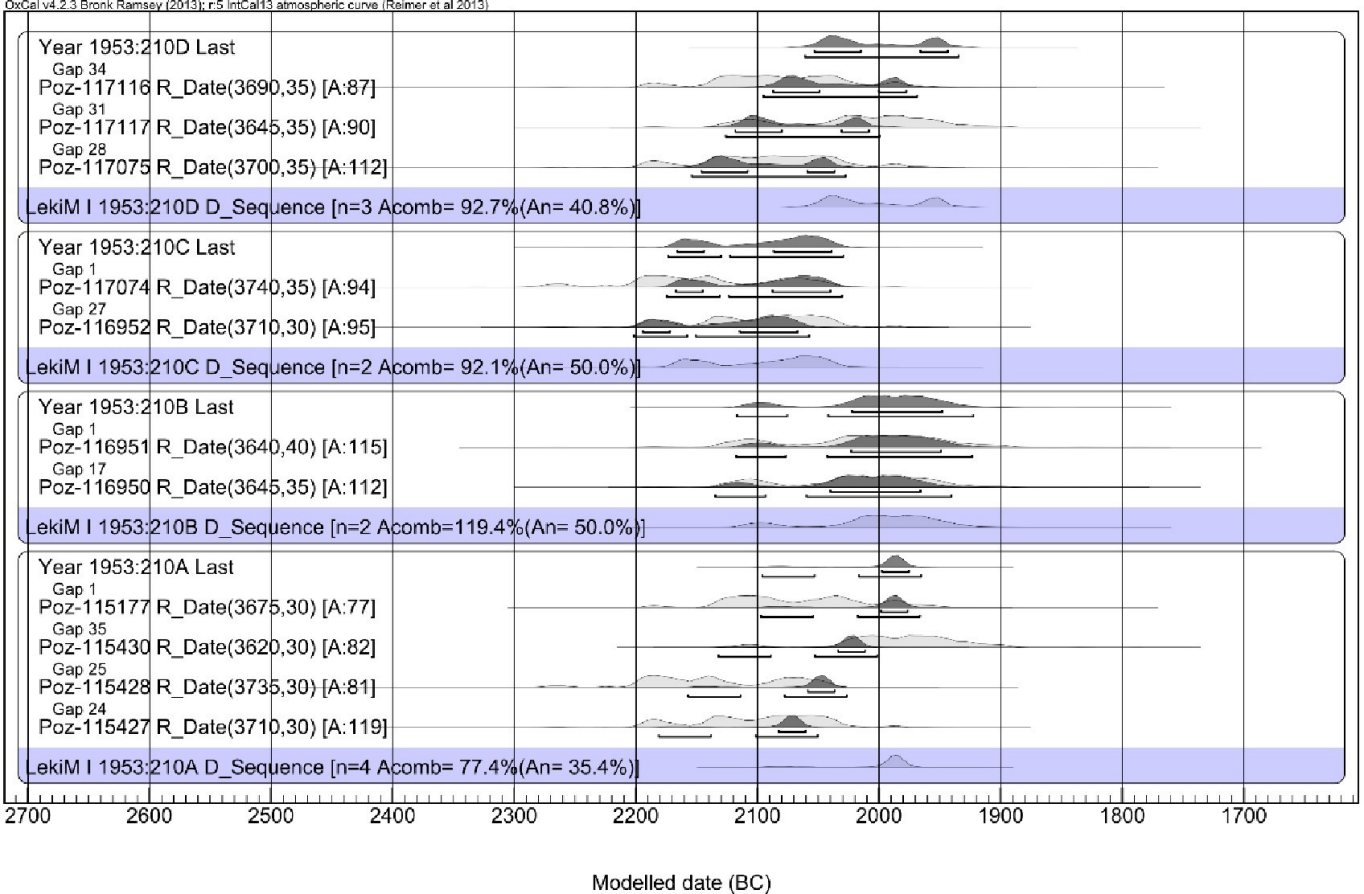
Łęki Małe, Barrow I. Calibration results of radiocarbon dates. The dated samples were bound in four dendrochronological sequences, so the differences in calendar dates of consecutive samples from a given sequence were known. The probability distribution of the date of the youngest increment associated with that sequence ("Last") is also shown for each sequence.

In the case of Barrow I, the preservation of charred wood from the central tomb allowed dendrochronological analysis and wiggle matching of the ^14^C determinations (Table 9; Fig 14), significantly narrowing the date range for each sample. In the dendrochronological analysis following ranges were obtained: for ŁM_A 1999–1676 BC, for ŁM_B 2024–1950 BC, for ŁM_C 2167–2145 BC and 2087–2040 BC, and for ŁM_D 2054–2016 BC and 1967–1944 BC (all ranges are of 68.2% probability) (Table 10).

**Table 10:** Łęki Małe, Barrow I. Results of calibration of the ^14^C age of samples: ŁM_A, ŁM_B, ŁM_C and ŁM_D. Wiggle matching modeling: OxCal v 4.4.2 [41], calibration curve IntCal 20 [42].

Sample code	Sequence code (OxCal)	Calibration of the ^14^C age ‐ wiggle matching modeling; dates BC			
		68.3% of probability (1σ)		95.4% of probability (2σ)	
		from	to	from	to
ŁM_A	D_Sequence LEKI MALE_A	1998	1977	2100	2054
				2019	1967
ŁM_B	D_Sequence LEKI MALE_B	2025	1948	2121	2075
				2046	1925
ŁM_C	D_Sequence LEKI MALE_C	2167	2146	2174	2131
		2087	2039	2121	2030
ŁM_D	D_Sequence LEKI MALE_D	2054	2017	2062	1937
		1966	1944		

**Fig 15:**
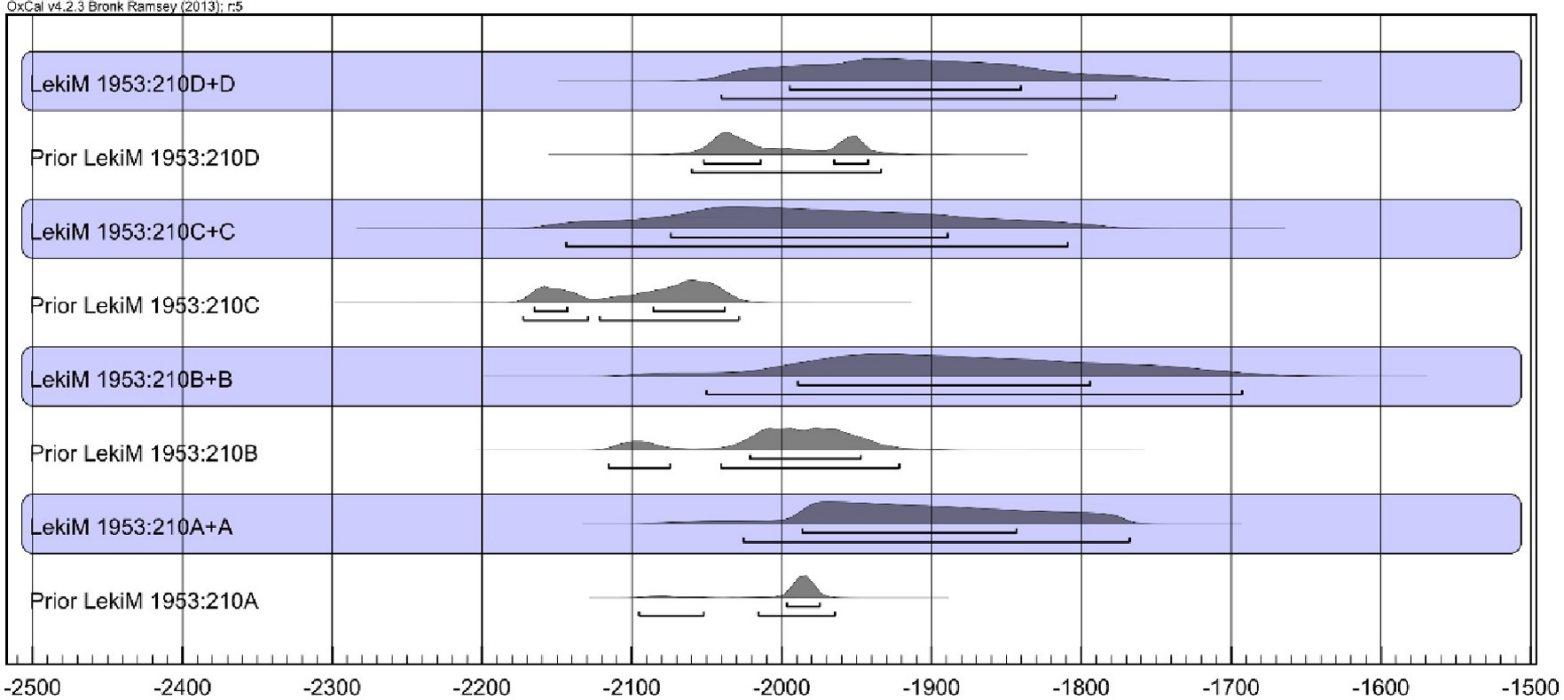
Łęki Małe, Barrow I. Probability distributions of the dates of the wood samples and (against a blue background) the dates of harvesting the trees from which the samples were derived. The time interval between the formation of the dated sample wood and the felling of the tree was assumed to be exponential and within the range assuming that the trees had a maximum of 300 annual increments.

**Fig 16:**
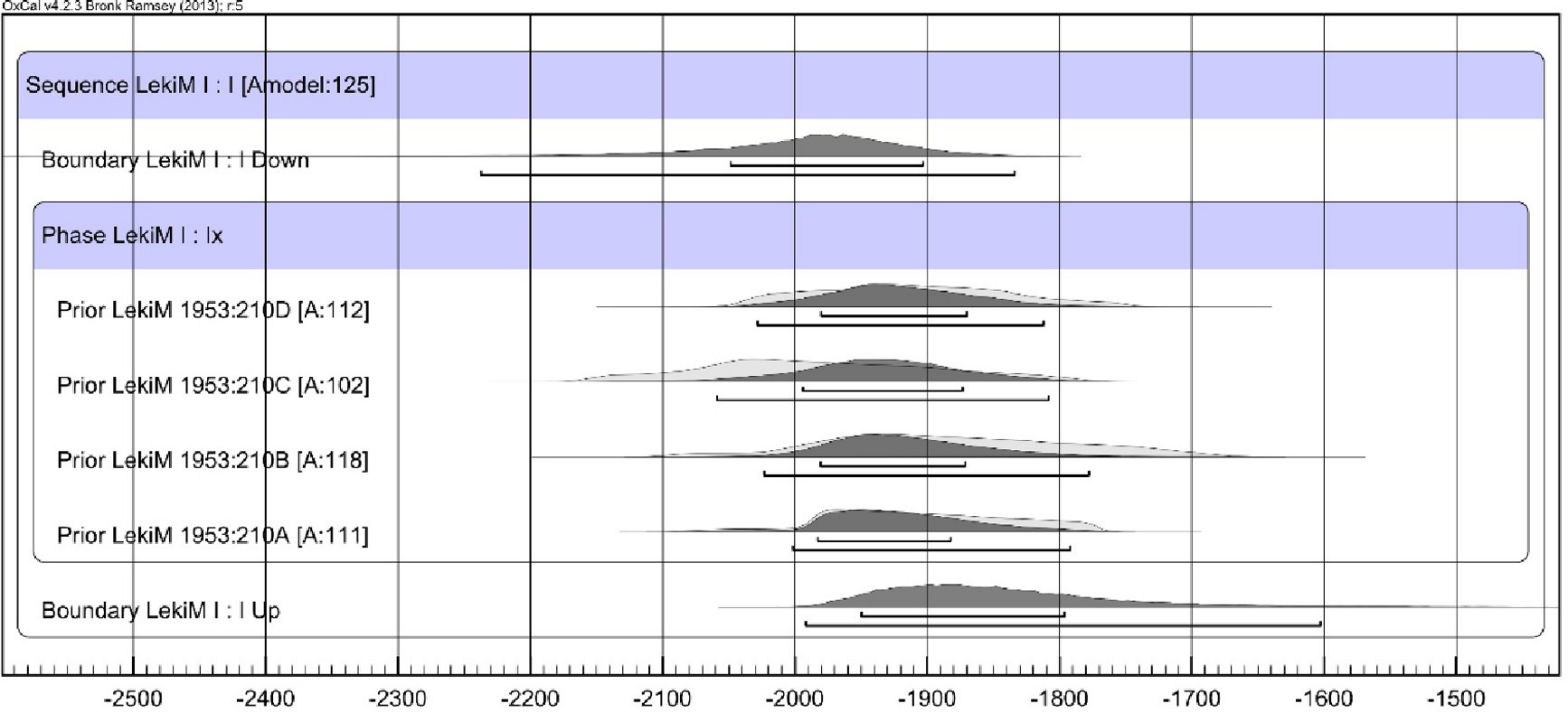
Łęki Małe, Barrow I. Results of Bayesian date set modeling. It was assumed that the dates of felling the trees (from which the tested wood came from) fall within one phase.

However, none of the samples included a bark or waney edge or sapwood, suggesting the impact of the old wood effect. In all likelihood, the number of missing tree rings was different for each sample. Nevertheless, we argue that the examined wood was used as timber to build the tomb chamber and therefore likely was selected from old trees. It was accepted that these were oaks with age up to 300 years (in Central European conditions, trunks of 300-year-old oak trees typically tend to be about 85 centimeters in diameter) [43,44]. Based on this assumption, we calculated the probable dates of felling for all trees (Fig 15). In addition, we assume that all trees were felled within one phase as they were all used in the construction of the same monument (”Phase”, Fig 16). The resulting Bayesian modeling suggests that the trees were felled around 2000/1900 BC or immediately after.

### Chronological sequence of barrows

In summary, using chronometric dating we reconstructed the date of construction of each tomb from each barrow, despite the fact that the earliest dating of the barrows derives from a different context in each case.

For Barrow IV the earliest dates are associated with level II; the un-dated level I might be even older. However, the stratigraphic information suggests there was no large time gap between the dated level II burials and the construction of the central tomb.

In Barrow III the situation is more problematic, as the oldest dates derive from fireplaces in level IV. The other dates belong to level VI, which is associated with the looting of the central tomb. In addition, we were able to date contexts from level VI associated with the looting of the central tomb. From other sites (e.g. Gemeinlebar, Franzhausen, Fidvár by Vráble) we know that Early Bronze Age looting took place not much after the construction of the central tomb [45–50]. It is, therefore, likely that the construction of the tomb took place less than two generations before the looting activities.

In Barrow II, ^14^C data on the human and animal bones indicate that it could have been built around 2000/1900 BC. For Barrow I we argue that the dated trees were part of the central wooden chamber and thus could be associated with the construction of the central tomb which may have been around 2000/1900 BC. Thus, except for Barrow III, there is a high probability that we caught the earliest construction period for each barrow with deviations that do not contradict the below proposed model.

**Fig 17:**
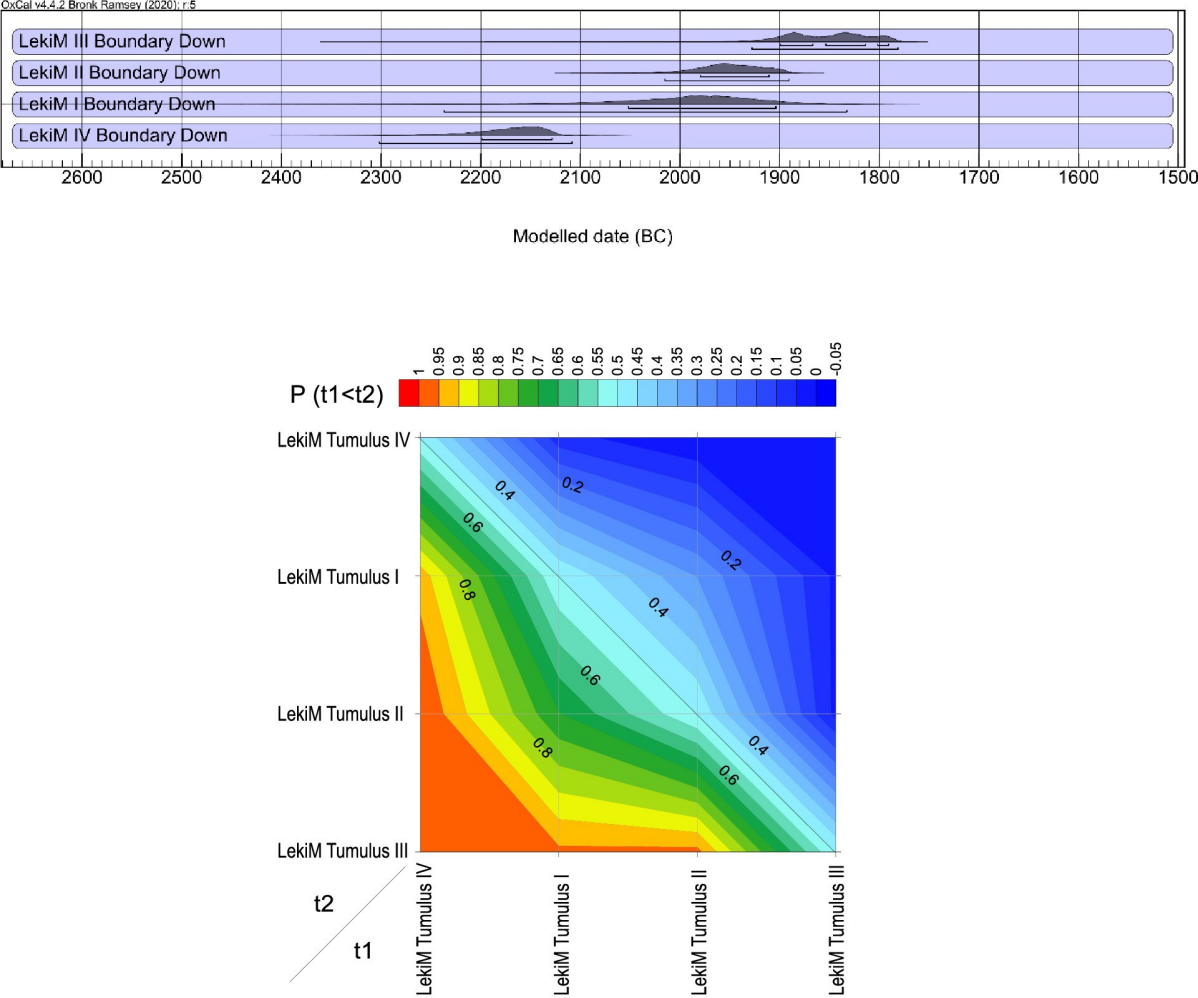
Łęki Małe. Comparison of the dates of the beginning of the use of barrows I-IV. Top: Probability distributions of the calendar dates of the lower bounds of Bayesian chronological models. The burial mounds were ranked according to the median distribution. Bottom: Diagram showing the probability that the beginning of the use of the burial x (with the date denoted by "t1") is older than that of the burial y (the date denoted by "t2").

**Fig 18:**
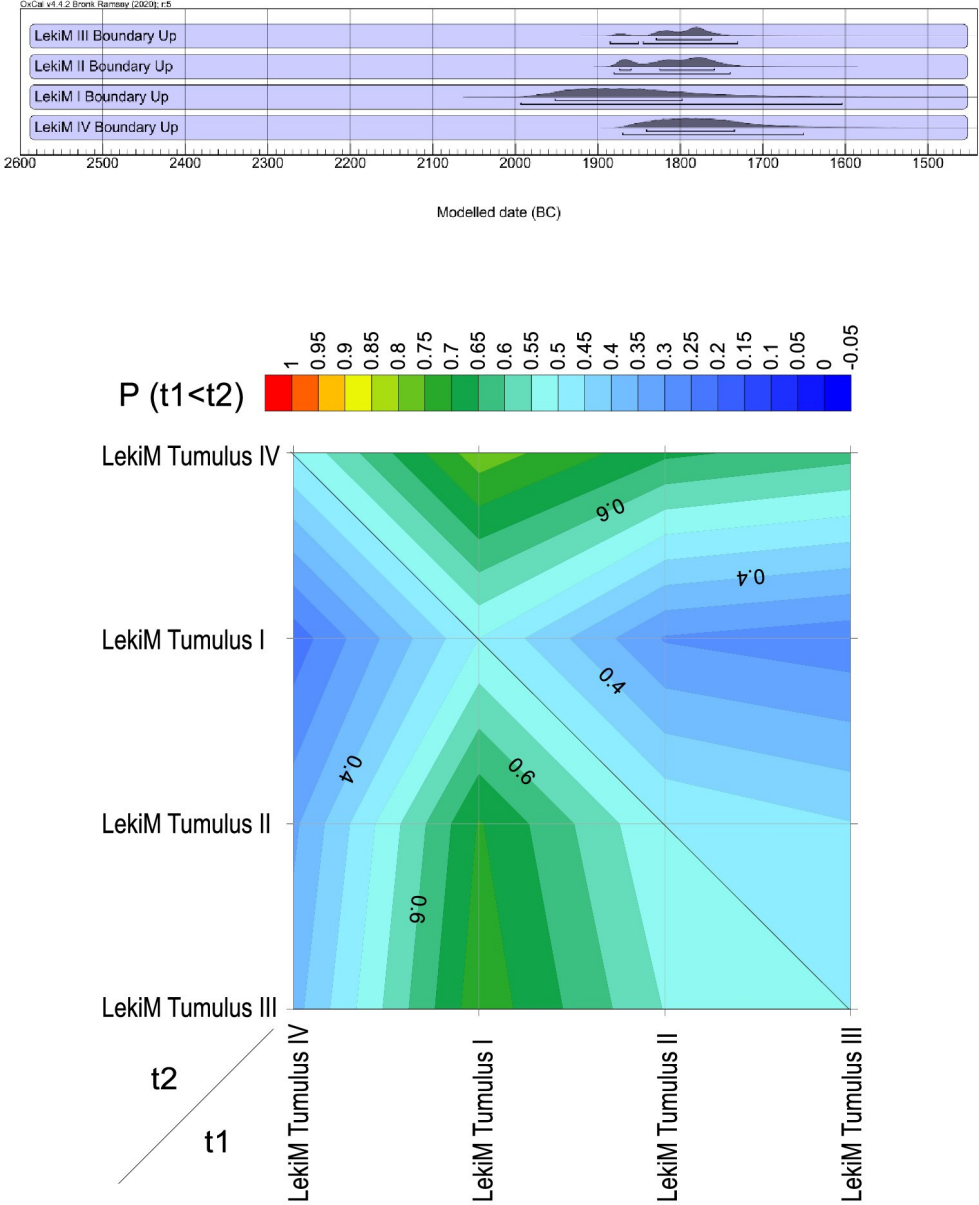
Łęki Małe. Comparison of the dates of the ends of the use of barrows I-IV. Top: Probability distributions of the calendar dates of the upper bounds of Bayesian chronological models. The burial mounds were ranked according to the median distribution. Bottom: Diagram showing the probability that the end of the use of the burial x (with the date denoted by "t1") is older than that of the burial y (the date denoted by "t2").

In conclusion, the analysis of absolute chronology (Fig 17) shows Barrow IV was raised first, around 2130–2120 BC. Some 100 years later Barrow I was erected. Shortly thereafter (20–30 years, one generation), Barrow II was built, and probably later (60–70 years, two generations) Barrow III. Early Bronze activities in the cemetery ceased about 1800/1775 BC, as evidenced by the dating of level VI in Barrows IV, II and III (Fig 18).

## The artefacts typo-chronology of Łęki Małe

The typo-chronological analysis of ceramics and metals from the barrows provide additional information regarding their relative chronology.

### Ceramic typo-chronology

Table 11 presents the results of the typo-chronological study of the pottery from the barrows. We found typological correlations with almost all phases of the Únětice periodization, except for the oldest proto-Únětice stage. Most types and examples of pottery correspond to the Únětice classic and post-classic phases, ca. 2000–1650 BC. Ceramic type was identified following Zich [51] and Kneisel, Schilz [52].

**Table 11:** Łęki Małe. Typological assessment of individual vessels.

No.	Barrow	Unit	SL	Types of vessels															References
				6G3	7H2	1A3	1A2	1C1	20B2 = GTB	12A1 [51] Łęki Małe/ Przecławice	3C2 = W3B	1A (gen.)	GO 05A	7K1	7K2	crucible	10B2	miniature	
1	IV	cluster 3	I	x*															[16]
2	IV	grave B	V				X												[16]
3	IV	grave B	V							x									[16]
4	IV	cluster 2	V										x						[16]
5	IV	quarter III	V								x								[16]
6	I	grave A/SE	I							x									[16]
7	I	grave A/SE	I								x**								[16]
8	I	grave A/NW	I		x														[16]
9	I	grave A/NW	I							x									[16]
10	I	grave B	IV					X											[16]
11	I	grave B	IV															x	[16]
12	I	grave D	V							x									[16]
13	I	grave D	V								x***								[16]
14	I	grave D	V								x***								[16]
15	I	grave D	V									x							[16]
16	I	grave D	V													x			[16]
17	I	cluster 1	V							x									[16]
18	I	cluster 1	V											x					[16]
19	I	cluster 2	V						x										[16]
20	I	cluster 3	V						x										[16]
21	I	cluster 4?	?														x*		[16]
22	I	grave C	V							x*									[16]
23	II						X												[16]
24	II														x				[16]
25	III	grave A	I							x									[16]
26	III	grave B	V			x													[16]
27	III	grave B	V											x					[16]

Notes: * the closest, ** with a handle, *** with feet, SL ‐ stratigraphic level, gen.–generally.

We distinguished six chronological/functional groups:

1. The old-Únětice phase: type 6G3 [51]. Analogy: Mikulovice, grave 95 [53] (Type X, but in Mikulovice without ornamentation).
2. The old/pre-classic Únětice phase: type 7H2 [51]. Analogy: Mikulovice, grave 1 [53] (closest to type K).
3. The pre-classic/classic Únětice phase, types 1A2 [51]. Analogy: Mikulovice, grave 18, type F; 1A3, 1C1 and 12A1 [51]. Analogy: Mikulovice, pits no. 2023 and 2412 [53] (closest to type K).
4. The classic/post-classic Únětice phase, types 1A, 3C2 [51]. Analogy: Mikulovice, grave 67 [53] (closest to type L), 20B2 [51], GO 05A [52]
5. The local forms, types 7K1, 7K2 [51]
6. The functional forms, type 10B2 [51], a miniature vessel and a crucible

The results mentioned above were used to develop a presence/absence seriation matrix indicating the date of use of each barrow according to the typo-chronological data (Table 12). The pottery from Barrow IV is the earliest, followed by that from Barrow I. The pottery from Barrows II and III is younger and the barrows are close together in time.

**Table 12:** Łęki Małe. Typochronology of pottery from barrows I–IV.

Barrow	Group 1	Group 2	Group 3	Group 4	Group 5	Group 6
IV	X		X	x		
I		X	X	x	x	x
II			X		x	
III			X		x	

### Metal typo-chronology

When considering the chronology of metal finds from the site of Łęki Małe, the most important are those from the destroyed Barrow VI and from the graves in Barrow I (Table 13). The largest collection of metal objects comes from Graves A and D in Barrow I, and the objects are diversified both in terms of form and raw materials. In addition to numerous bronze objects, gold rings made of wire with a return coil (*Noppenringe*) were found in both graves.

**Table 13:** Łęki Małe. Register of metal artefacts.

Barrow	Grave	Noppenringe (gold)	Halberd	Dagger	Axe	Massive bracelet	Awl	Cypriot pin	Eyelet pin
I	A	2	1	1	1	2		1	
I	D	3		1	1		1		2
III	B	2							
VI	-								1
Total		7	1	2	2	2	1	1	3

In total, seven gold *Noppenringe* were discovered at the site: two specimens in Barrow III Grave B, two in Barrow I Tomb A, and three in Barrow I Grave D [10,14]. This ornament form is characteristic of the Únětice, but recorded also outside its area of strict occurrence [54] where it often signals the presence of a greater amount of “Únětice influences” in material assemblages [55]. These finds, due to their extensive distribution throughout Central Europe, differ in chronological position. From the point of view of the examples examined, however, the most important seems to be their presence in the hordes of Únětice, often accompanied by other metal forms also known from Łęki Małe, e.g. eyelet pins (*Ösenkopfnadel*). Taking into account the available radiocarbon dating of the *Ösenkopfnadel* (see below), the gold *Noppenringe* from Łęki Małe likely belong to the classic Únětice phase, i.e. the BA2a period, 2000–1750 BC [53,54].

In principle, all bronze artefacts discovered on the site have a similar chronological position. Typologically, all of the noted forms are associated with the classic Únětice phase. On the basis of the existing information, it seems possible, however, to separate older and younger finds within this phase, and thus also to indicate a potential chronological difference between Tomb A and Grave D from Barrow I.

In the collection of finds from Tomb A, the M1a type halberd and the Cypriot pin constitute potential evidence of the earlier (within the classic Únětice phase) chronological position of the Tomb A. The Cypriot pin is a form with a relatively long tradition of use, noted already in the early Únětice stage and in the earlier Nitra stage [56,57]. Together with the aforementioned M1a halberd, this set of objects is characteristic for the period around 2100–1950 BC [58]. The remaining bronze objects discovered in Tomb A do not allow further refining of the absolute chronology of the burial. It should be emphasized, however, that the Cypriot pin from Tomb A Barrow I is only partially preserved. Its classification to the Cypriot type was determined by the preserved fragment of the wire coil in the upper part of the pin, but the pin head was not preserved. Therefore, this pin could also potentially have been of a form similar to the pin from the Prague-Miškovice grave 18 [59], radiocarbon dated to approx. 1970–1740 BC (95.4% probability) [60] i.e. in the period of the occurrence of the eyelet pins [59].

The two pins found in Grave D Barrow I are the most chronologically sensitive finds. Although both specimens are currently fragmentary, an analysis of the archive drawing documentation allows for careful identification of the two different types of eyelet pins represented. The first specimen was decorated with three grooves in the upper part. There is a preserved “eye” on the head of this pin fragment. The second specimen does not have a preserved “eye”, and its upper part is decorated with a herringbone ornament [61]. The results of research on the absolute chronology of Early Bronze Age burials equipped with eyelet pins allow us to determine the age of the specimens from Grave D for the period around 1950–1750 BC [53,60]. Furthermore, most of the published dating of Czech finds point to the beginning of this period (1950–1850 BC) as the stage of the most frequent use of the eyelet pins [59].

An additional eyelet pin comes from the heavily destroyed Barrow VI. It is an unornamented specimen, preserved in its entirety, with an “eye” stretched between the edges of the pin head [16]. Specimens with a similar form are known from a large collection of finds from the Mikulovice site in Czech (23 such pins discovered in 20 graves [53]). The chronological position of the decorated specimens is specific: among the radiocarbon-dated burials equipped with this form of pins, there are both finds dated around 1900 BC, and much later, around 1750 BC [53]. Similar results have been obtained for finds from the Circum-Harz group. In this case, based on the analysis of available radiocarbon dates, the existence of three groups of eyelet pins with different chronological placement was suggested [62]. The specimen from Barrow VI can be included in the chronologically youngest Group 3 dated to the period 1900–1750 BC [62].

Taking into account the above information, the hypothesis can be put forward that Tomb A Barrow I is older than Grave D Barrow I and that both are older than Barrow VI. As discussed earlier, in the case of Barrow I this interpretation is further supported by both the stratigraphic data and the analysis of the ^14^C dating.

### Conclusion: Relative chronology of Łęki Małe barrows

The typo-chronology of the pottery in Łęki Małe barrows supports the results obtained from the absolute chronological data. The sequence of the pre-classic-classic phase is visible in the case of Barrow I, while the pre-classic phase is limited to Barrow IV. The spatial shifts from the larger Barrow IV to Barrow I and then to Barrows II and III, could indicate a decisive change in the biography of the cemetery: the richly furnished Barrow I became the new focal area on which the social development was oriented.

The metal finds generally date to the first three centuries of the 2nd millennium BC. They provide the vital evidence that Barrow VI was built later than Barrow I.

### The general biography of the Łęki Małe cemetery

The Łęki Małe cemetery includes at least fourteen burial mounds, with the oldest (Barrow IV) located on its south-eastern fringe, closest to the intersection of the Mogilnica and the Obra Rivers. Accordingly, the expansion of the cemetery proceeded up the Mogilnica River in a north-western direction.

**Fig 19:**
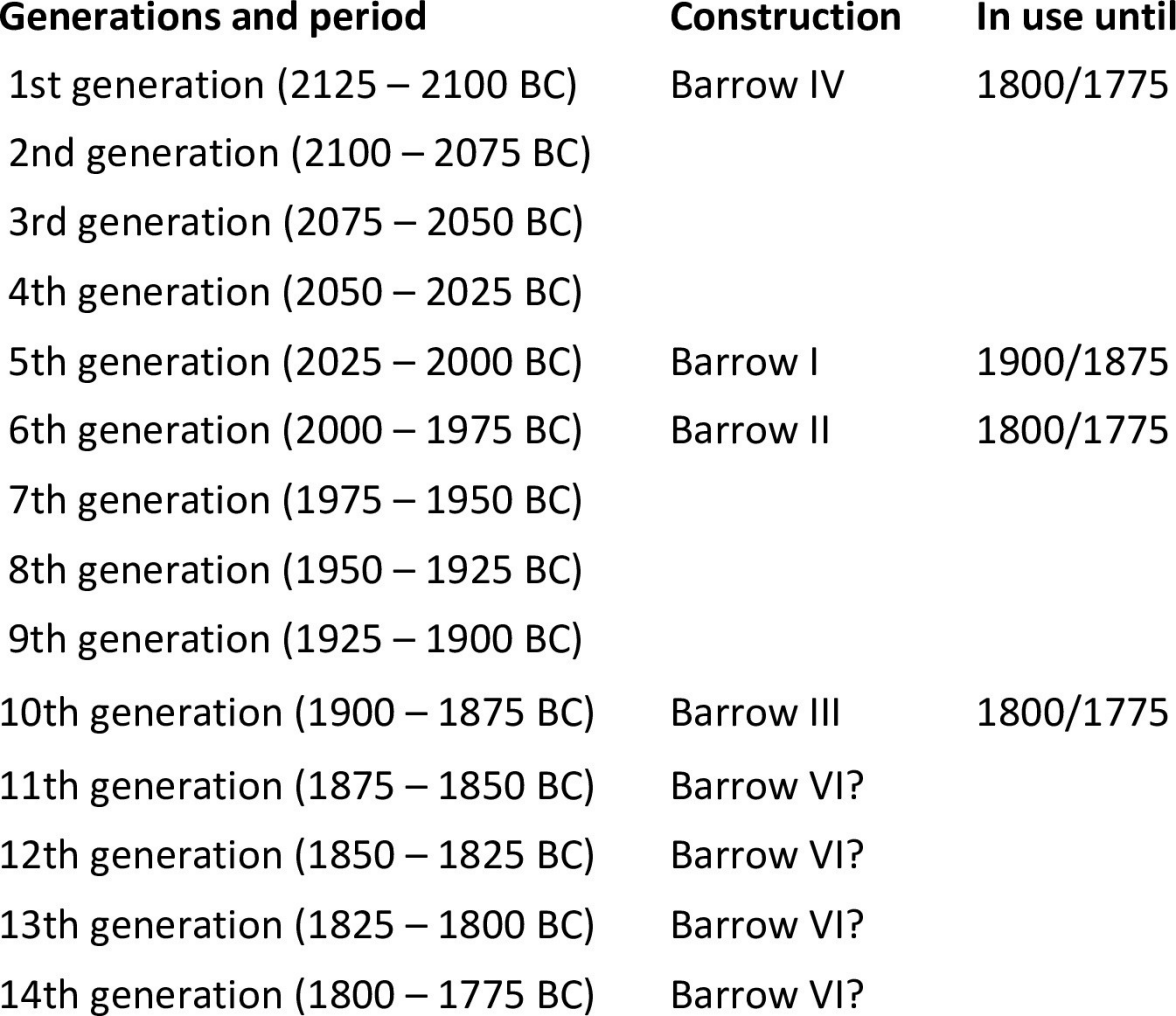
Łęki Małe. Beginnings and ends of barrows use in the cemetery against possible human generations.

**Fig 20:**
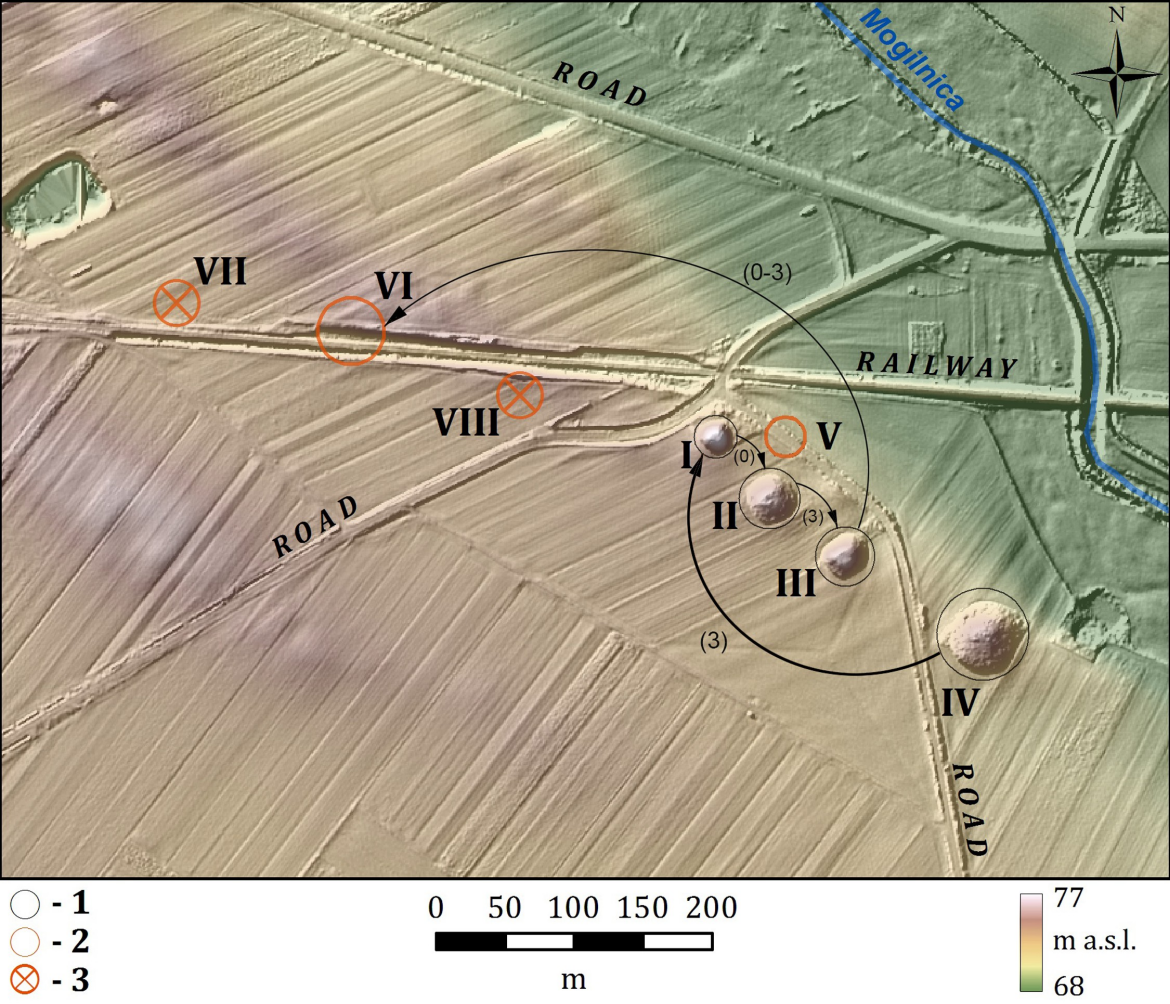
Łęki Małe. Sequence of construction of barrows in the cemetery. Key: 1 –barrows excavated and then reconstructed; 2 –barrows partially preserved, 3 –barrows registered in the mid-twentieth century.

In the Early Bronze Age, the cemetery was in use from 2130/2120 to 1800/1775 BC (Figs 17 and 18). One possible assumption is that one barrow was raised per generation. If we combine this with the information that fourteen burial mounds were in existence in 1881 (Table 1), the following sequence of construction and use of barrows can be proposed (Fig 19). It should be emphasized that this model is schematic. Barrows were unlikely to be built with such regularity, though the average rate of building of mounds could have been very close to what was proposed above. Although the cemetery expanded to the north-west, it was not a simple expansion where each mound arose in a line, as shown by the older portion of the cemetery (Fig 20).

### The Łęki Małe barrow cemetery and other Únětice “princely” mounds

Three regions in central Europe display the most impressive Únětice “princely” barrows: the Circum-Harz group (Bornhöck, Helmsdorf, Leubingen), Silesia (Kąty Wrocławskie and Szczepankowice) and Greater Poland (Łęki Małe and the Krotoszyn Forest, barrow no. 35) [63]. All three belong to the northern part of the Únětice distribution area. However, it is essential to remember that the „princely” barrows phenomenon did not just confine itself to these few regions. More information is available about the sites where burial mounds once existed. There are archival records preserved, proving their presence or the finds suggesting that the mounds used to be there [63–66]. This was also the case of the exceptional mound of the Bornhöck, where only the archival sources helped in the reconstruction of a nowadays flat site [67].

Unlike the other sites mentioned above, each of which contains a single burial mound, the Łęki Małe cemetery is composed of more than ten barrows. The discussed data correspond to use over ca. 350 years or 14 human generations. In addition to having a long duration of use, burial activities at Łęki Małe begin in the second half of the 22^nd^ century, much earlier than at other Únětice barrow sites. The earliest Łęki Małe burial mounds predate other known Únětice barrows (Leubingen, Szczepankowice and Kąty Wrocławskie) by at least one and a half century (Table 13). In Greater Poland, not only Łęki Małe Barrows IV, I and II, but also burial mound no. 35 in the Krotoszyn Forest [63] confirm the early use of barrows by Únětice communities (Table 14). All other well-dated Únětice “princely” barrows fit within the time range covered by the Łęki Małe barrows, but no other site displays grave mound building activities for longer than one generation.

**Table 14:** Beginnings of barrows in the Únětice area and basis for their chronological assessment.

Barrow	Únětice group	Beginning (BC)	Basis of dating	References
Łęki Małe, Barrow IV	Greater Poland	around 2130	^14^C	this paper
Łęki Małe, Barrow I	Greater Poland	2000–1975	^14^C and dendro	this paper
Krotoszyn Forest, Barrow 35	Greater Poland	1976–1915	^14^C	[63]
Łęki Małe, Barrow II	Greater Poland	1975–1950	^14^C	this paper
Leubingen	Circum Hercynian	1942	Dendro	[68,69]
Szczepankowice	Silesia	1937–1889	^14^C	[70]
Kąty Wrocławskie	Silesia	1951–1765	^14^C	[71]
Łęki Małe Barrow III	Greater Poland	1900–1800	^14^C	this paper
Helmsdorf*	Circum Hercynian	1829/28	Dendro	[67]
Bornhöck	Circum Hercynian	around 1800	^14^C	[68]

*Cf. additional information concerning ^14^C datings see [72].

With the new chronological results for Łęki Małe, the ritual practice of erecting “princely” barrows can no longer be considered only a late Únětice phenomenon, as earlier proposed [68]. The oldest Łęki Małe barrow was already erected around 2130 BC. The next youngest known Únětice barrow is from Leubingen and dendrochronological dates place its construction in the mid-20th century BC (Table 14), around seven generations later.

The proposed duration of ritual Únětice practices at Łęki Małe (ca. 2125–1775 BC) is contemporary with the Únětice fortified settlement Bruszczewo (ca. 2125–1650 BC) located ca. 15 km to the south. J. Müller and J. Kneisel argue that Bruszczewo was a regional center, particularly as regards metallurgy [73]. The presence of both a fortified center and an extraordinary barrow cemetery strongly indicate that a new socio-political system was established in the Kościan area during the Early Bronze Age. The regional pollen profile from Lake Wonieść suggests a rapid increase in forest clearance at the same time, beginning at the latest by the 22nd century BC [74].

The existence of stratified Únětice socio-political systems has already been suggested. For the Kościan area, a socio-political system with a stratified society was reconstructed by Czebreszuk [75]. For the Circum-Harz group an even more differentiated and complex socio-political system was reconstructed wherein “princely”barrows are seen to be indicators of social hierarchies [68,76,77].

The early dating of the Łęki Małe cemetery demonstrates the existence of comparable, complex social structures since Únětice beginnings and its duration at least 14 generations. In contrast to the Circum-Harz group, where the origins of social differentiation (around the 19th-18th century BC) are found in early Únětice development, in Greater Poland the roots of social differentiation stretch back into the preceding Late Neolithic societies. Furthermore, our new early dates for “princely” barrows, coinciding with the very beginning of Únětice’s independent development ‐ close the time gap between this type of funeral rite in Únětice and the funeral rite of burial mounds of the Corded Ware phenomenon [68,78,79].

In the Polish Lowlands, the collected ^14^C dates indicate the development of the Corded Ware phenomenon lasted until ca. 2200 BC [64,80]. Thus, in Greater Poland, Únětice communities may have directly adapted the barrow concept from Corded Ware social practices [79]. Apart from the chronological proximity, there is a continuity of use of the area in Łęki Małe: a flat grave belonging to the late Corded Ware phase was documented and, at the place of Barrow IV, relatively long-lasting activity of the Corded Ware community has been proven [18]. Similarly, in the Circum-Harz group the “princely” barrow of Helmsdorf was placed upon a Corded Ware burial mound [81]. It is therefore very likely that in both regions the long lasting tradition of using burial mounds to encourage the ideology of the social hierarchy was transferred from Final Neolithic communities to Early Bronze Age societies.

## Conclusions

This article focused on new data on the chronology of the cemetery at Łęki Małe. This data provides a much stronger database and rich body of evidence that calls for close analysis and interpretation in order to offer a new model of the European past during the late 3rd and the early 2nd millennium BC. At this point, we will only highlight some of the research areas we plan to focus on in future publications.

Łęki Małe has provided significant new insights into the development of Únětice society in Greater Poland. Around 2150 BC, simultaneously with increased agrarian activities, leading to an increased clearing of the landscape [74], and at the same time as the establishment of the fortified settlement of Bruszczewo [73], which represents the regional distribution center for supra-regional products and metal production, the series of large burial mounds in Łęki Małe was established in the landscape.

The uniqueness of the Kościan region can certainly be traced back to its controlling role in the amber exchange between the Baltic region and Central Europe, as well as probably with the Aegean [82,83], and also to its role in the development of metal casting technology. The latter can be seen particularly in the early dating for overlay casting a pin in Bruszczewo [73] and a casting of a hollow core dagger found in Przysieka Polska [65].

The “princely” burial mounds suggest that permanent or stable structures of power (units of political organization) existed in the Únětice world. Previously, it was argued that this type of political organization lasted for at least 150 years (1950–1800 BC). Thanks to our research, we now know that they developed at least twice as long, i.e. about 330 years (2130–1800 BC). This extends the duration of the politically complex Únětice society from six/seven generations, to possibly fourteen generations. The mechanisms that stabilized the political world of Únětice for so many generations should be the subject of in-depth research in the future.

As it is unlikely that the new social display of powerful people in the form of “princely” barrows would have developed autonomously within the Kościan group, these communities probably followed patterns that also emerged in other Únětice regions: especially on the Saale and in Silesia, but probably also in Bohemia and Moravia. In other words, throughout this long period, the structures in question were not only subjects of internal (Únětice) political life. They also influenced other centers of Europe, shaping the political and cultural face of our continent.

## References

[1] Müller J, Czebreszuk J. Bruszczewo–ein frühbronzezeitliche Siedlung mit Feuchtbodenerhaltung in Grosspolen. Vorbericht zu den Ausgrabungen 1999–2001. Germania. 2003;81(2):443–480.

[2] Jaeger M, Czebreszuk J. Does a Periphery Look Like That? The Cultural Landscape of the Unetice Culture’s Kościan Group. In: Landscapes and Human Development: The Contribution of European Archaeology. Universitätsforschungen zur prähistorischen Archäologie 191. Bonn: Dr. Rudolf Habelt GmbH; 2010. p. 217–236.

[3] Czebreszuk J, Müller J, editors. Ausgrabungen und Forschungen in einer prähistorischen Siedlungskammer Großpolens/Badania mikroregionu osadniczego z terenu Wielkopolski. Band I/ Część I. Forschungsstand–Erste Ergebnisse–Das östliche Feuchtbodenareal/Stan badań –Pierwsze wyniki–Wschodnia, torfowa część stanowiska. Studien zur Archäologie in Ostmitteleuropa/Studia nad Pradziejami Europy Środkowej 2. Poznań-Kiel-Rahden/Westf.: Verlag Marie Leidorf GmbH; 2004.

[4] Czebreszuk J, Müller J, editors. Bruszczewo III. The settlement and fortification in the mineral zone of the site. Studien zur Archäologie in Ostmitteleuropa/Studia nad Pradziejami Europy Środkowej 13. Poznań-Bonn: Dr. Rudolf Habelt GmbH; 2015.

[5] Müller J, Czebreszuk J, Kneisel J, editors. Bruszczewo II. Ausgrabungen und Forschungen in einer prähistorischen Siedlungskammer Grosspolens/Badania mikroregionu osadniczego z terenu Wielkopolski. Studien zur Archäologie in Ostmitteleuropa/Studia nad Pradziejami Europy Środkowej 6. Bonn: Verlag Dr. Rudolf Habelt GmbH; 2010.

[6] Silska P. Wczesnobrązowa osada obronna w Bruszczewie. Badania 1964–1968. Bibliotheca Fontes Archaeologici Posnanienses 13. Poznań: Muzeum Archeologiczne w Poznaniu; 2012.

[7] Kneisel J. The Problem of the Middle Bronze Age Inception in Northeast Europe–or: Did the Ŭnětice Society Collapse? In: Kneisel J, Kirleis W, Dal Corso M, Taylor N, Tiedtke V, editors. Collapse or Continuity? Enviroment and Development of Bronze Age Human Landscapes. Universitätsforschungen zur prähistorischen Archäologie 205. Bonn: Dr. Rudolf Habelt GmBH; 2012. p. 209–233.

[8] Müller J. 1600 B.C.–Social topographies and the development of Early Bronze Age societies in Central Europe. In: Meller H, Bertemes F, Bork HR, Risch R, editors. 1600 –Kultureller Umbruch im Schatten des Thera-Ausbruchs? 4. Mitteldeutscher Archäologentag vom 14. bis 16. Oktober 2011 in Halle (Saale). Tagungen des Landesmuseums für Vorgeschichte Halle 9. Halle: Landesmuseum für Vorgeschichte Halle (Saale); 2013. p. 527–537.

[9] Czebreszuk J, Müller J, Jaeger M, Kneisel J, editors. Bruszczewo IV. Natural resources and economic activities of the Bronze Age people. Studien zur Archäologie in Ostmitteleuropa/Studia nad Pradziejami Europy Środkowej 14. Poznań-Bonn: Dr. Rudolf Habelt GmbH; 2015

[10] Kowiańska-Piaszykowa M, Kurnatowski S. Kurhan kultury unietyckiej w Łękach Małych, pow. Kościan. Fontes Archaeologici Posnanienses 1954;4:43–76.

[11] Schwartz W. Materialien zu einer prähistorischen Karte der Provinz Posen. Band III. Posen; 1881

[12] Kostrzewski J. Kurhany kultury unietyckiej w Małych Łękach, w pow. kościańskim. Polska Akademia Umiejętności. Sprawozdania z czynności i posiedzeń, Rok 1934. 1935;39(3):25–26.

[13] Kowiańska-Piaszykowa M. Wyniki badań archeologicznych kurhanu III kultury unietyckiej w Łękach Małych, w pow. kościańskim. Fontes Archaeologici Posnanienses. 1957;7:116–138.

[14] Kowiańska-Piaszykowa M. Wyniki badań archeologicznych kurhanu IV kultury unietyckiej w Łękach Małych, pow. Kościan. Fontes Archaeologici Posnanienses. 1968;19:6–31.

[15] Kowiańska-Piaszykowa M. Badania wykopaliskowe na kurhanie VI w Łękach Małych, pow. Kościan w 1970 r. Fontes Archaeologici Posnanienses. 1971;22:206–208.

[16] Kowiańska-Piaszykowa M. Cmentarzysko kurhanowe z wczesnej epoki brązu w Łękach Małych w Wielkopolsce. Bibliotheca Fontes Archaeologici Posnanienses 12. Poznań: Muzeum Archeologiczne w Poznaniu; 2008.

[17] Szmyt M, Czebreszuk J. Before Barrows. Funnel Beaker culture settlement preceding the Early Bronze Age cemetery in Łęki Małe (site Wilanowo 12). In: Dębiec M, Górski J, Müller J, Nowak M, Pelisiak A, Saile T, Włodarczak P, editors. From Farmers to Heroes? Archaeological Studies in Honor of Sławomir Kadrow. Universitätsforschungen zur prähistorischen Archäologie 376. Bonn: Dr. Rudolf Habelt GmbH; 2022. p. 337–346.

[18] Czebreszuk J, Szmyt M. Before Barrows: the activity of Corded Ware communities at the ‘princely barrow’ cemetery in Łęki Małe, Wielkopolska region. In preparation.

[19] Lipińska A. Grób kultury ceramiki sznurowej z Łęk Małych pow. Kościan. Fontes Archaeologici Posnanienses. 1963;13:311–313.

[20] Krzyszowski A. Rytualne paleniska czy obiekty grobowe (?) z przełomu okresu ’późnorzymskiego’ i wczesnych faz wczesnego średniowiecza w Wilanowie (stan. 12), gmina Kamieniec, w woj. wielkopolskim. Slavia Antiqua. 2010;51:165–225.

[21] Krzyszowski A. Kultfeuerstellen der römischen Kaiserzeit oder frühmittelalterliche Gräber des Typus Alt Käbelich–erste Ergebnisse der Ausgrabungen in Wilanowo, Fst. 12, Gem. Kamieniec, Woj. Wielkopolskie, in den Jahren 2006 und 2007. In: Jaszewska A, Michalak A, editors. VI Polsko-Niemieckie Spotkania Archeologiczne, Garbicz, 5–6 czerwca 2008. Biblioteka Archeologii Środkowego Nadodrza 4. Zielona Góra: Wydawnictwo Fundacji Archeologicznej; 2011. p. 169–187.

[22] Naumowiczówna E. Wczesnośredniowieczna jama w Łękach Małych, pow. Kościan. Fontes Archaeologici Posnanienses. 1963;13:325–328.

[23] Bartkowski T. Krajobraz okolicy Łęk Małych w epoce brązu. Fontes Archaeologici Posnanienses. 1954;4:77–86.

[24] Krzysztofka M. Objaśnienia do Szczegółowej Mapy Geologicznej Polski 1:50000. Arkusz Kościan. Warszawa: Państwowy Instytut Geologiczny; 1993.

[25] Hildebrandt-Radke I. Pradziejowa i wczesnohistoryczna antropopresja i jej zapis w środowisku przyrodniczym na przykładzie regionu środkowej Obry (Wielkopolska). Poznań: Bogucki Wydawnictwo Naukowe; 2013.

[26] Goslar T, Czernik J, Goslar E. Low-energy 14C AMS in Poznań Radiocarbon Laboratory, Poland. Nuclear Instruments and Methods in Physics Research B. 2004;223(4):5–11.

[27] Godycki M. Szkielety w kurhanie IV w Łękach Małych, pow. Kościan. Fontes Archaeologici Posnanienses. 1968;19:32–35.

[28] Allentoft ME, Sikora M, Sjögren K-G, Rasmussen S, Rasmussen M, Stenderup J, et al. Population genomics of Bronze Age Eurasia. Nature. 2015;522(7555):167–72. doi: 10.1038/nature14507 26062507

[29] Vogel JC, Waterbolk HT. Groningen Radiocarbon Dates X. Radiocarbon. 1972;14(1):6–110.

[30] Rassmann K. Zur Forschungsstand der absoluten Chronologie der frühen Bronzezeit in Mitteleuropa auf der Grundlage von Radiokarbondaten. Acta Archaeologica. 1996;67:199–209.

[31] Görsdorf J. 14C-Datierungen des Berliner Labors zur Problematik der chronologischen Einordnung der Bronzezeit in Mitteleuropa. In: Rassmann K, editor. Spätneolithikum und frühe Bronzezeit im Flachland zwischen Elbe und Oder. Beiträge zur Ur- und Fruhgeschichte Mecklenburg-Vorpommerns 28. Lübstorf: Archäologisches Landesmuseum Mecklenburg-Vorpommern; 1993. p. 97–117.

[32] Rewekant A. Analiza antropologiczna szczątków kostnych. Łęki Małe, kurhan IV. Unpublished manuscript; 2017.

[33] Warner RB A proposed adjustment for the ’old-wood effect’. In: Mook GW, Waterbolk HT, editors. Proceedings of the Second International Symposium 14C and Archaeology, Groningen 1987. Strasbourg: Council of Europe; 1990. p. 159–172.

[34] Wokroj FL. Materiał antropologiczny z kurhanu III w Łękach Małych, pow. kościański. Fontes Archaeologici Posnanienses. 1957;7:140–142.

[35] Krysiak K. Materiał kostny zwierzęcy z kurhanu III w Łękach Małych w pow. kościańskim. Fontes Archaeologici Posnanienses. 1957;7:142–143.

[36] Geyh MA. Bomb radiocarbon dating of animal tissues and hair. Radiocarbon. 2001;43(2): 723–730.

[37] Bronk Ramsey C. Bayesian analysis of radiocarbon dates. Radiocarbon. 2009;51(1):337–360.

[38] Bronk Ramsey C, Lee S. Recent and planned developments of the program OxCal. Radiocarbon. 2013;55(2–3):720730.

[39] Makarowicz P, Goslar T, Niebieszczański J, Cwaliński M, Kochkin IT, Romaniszyn J, Lysenko SD, Ważny T. Middle Bronze Age societies and barrow line chronology. A case study from the Bukivna ‘necropolis’, Upper Dniester Basin, Ukraine. Journal of Archaeological Science. 2018;95:40–51.

[40] Bronk Ramsey C. Radiocarbon calibration and analysis of stratigraphy: the OxCal program. Radiocarbon. 1995;37:425–430.

[41] Bronk Ramsey C. OxCal v4.4.2. Oxford (https://c14.arch.ox.ac.uk); 2020.

[42] Reimer PJ, Austin WEN, Bard E, Bayliss A, Blackwell PG, Bronk Ramsey C, et al. The IntCal20 Northern Hemisphere Radiocarbon Age Calibration Curve (0–55 cal kBP). Radiocarbon. 2020;62(4):725–757.

[43] Bugała W, editor. Dęby. Poznań: Bogucki Wydawnictwo Naukowe; 2000.

[44] Danielewicz W, editor. Dąbrowy krotoszyńskie. Monografia przyrodniczo-gospodarcza. Poznań: Oficyna Wydawnicza G&P, Gościański & Prętnicki; 2016.

[45] Bátora J. Problematika sekundárneho otvárania hrobov v kultúrach starsej doby bronzovej na juhozápadnom Slovensku. In: Kadrow S, editor. A turning of ages/Im Wandel der Zeiten: Jubilee book dedicated to Professor Jan Machnik on his 70th anniversary. Kraków: Institute of Archaeology and Ethnology Polish Academy of Science; 2000. p. 1–22.

[46] Kümmel C. Ur- und frühgeschichtlicher Grabraub: Archäologische Interpretation und kulturanthropologische Erklärung. Tübinger Schriften zur Ur- und Frühgeschichtlichen Archäologie 9. Münster: Waxmann; 2009.

[47] Müller-Scheeßel N, Bátora J, Gresky J, Reiter SS, Stucky K, Rassmann K. In search of the modus operandi: Reopenings of Early Bronze Age burials at Fidvár near Vráble, south-west Slovakia. In: Aspöck E, Klevnäs A, Müller-Scheeßel N, editors. Grave disturbances: The archaeology of post-depositional interactions with the dead. Studies in Funerary Archaeology 14. Oxford, Philadelphia: Oxbow; 2020. p. 189–205.

[48] Neugebauer JW. Die Nekropole F von Gemeinlebarn, Niederösterreich: Untersuchungen zu den Bestattungssitten und zum Grabraub in der ausgehenden Frühbronzezeit in Niederösterreich südlich der Donau zwischen Enns und Wienerwald. Römisch-Germanische Forschungen 49. Mainz am Rhein: Zabern; 1991.

[49] Randsborg K. Plundered Bronze Age graves: Archaeological & social implications. Acta Archaeologica. 1998;69:113–138.

[50] Sprenger S. Zur Bedeutung des Grabraubes für sozioarchäologische Gräberfeldanalysen: eine Untersuchung am frühbronzezeitlichen Gräberfeld Franzhausen I, Niederösterreich. Fundberichte aus Österreich: Materialhefte, Reihe A 7; Horn: Berger; 1999.

[51] Zich B. Studien zur regionalen und chronologischen Gliederung der nördlichen Aunjetitzer Kultur. Vorgeschichtliche Forschungen 20. Berlin-New York: De Gruyter; 1996.

[52] Kneisel J, Schilz Ch. Die Keramik aus dem östlichen Feuchtbodenareal. In: Czebreszuk J, Müller J, editors. Ausgrabungen und Forschungen in einer prähistorischen Siedlungskammer Großpolens/Badania mikroregionu osadniczego z terenu Wielkopolski. Band I/ Część I. Forschungsstand–Erste Ergebnisse–Das östliche Feuchtbodenareal/Stan badań –Pierwsze wyniki–Wschodnia, torfowa część stanowiska. Poznań-Kiel-Rahden/Westf.: Verlag Marie Leidorf GmbH; 2004. p. 153–246.

[53] Ernée M, Langová M, Langová M, Arppe L, Bednář P, Berger D, et al. Mikulovice. Pohřebiště starši doby bronzové na Jantarové stezce/Early Bronze Age Cemetery on the Amber Road. Památky archeologické, Supplementum 21. Praha: Archeologický ústav AV ČR; 2020.

[54] Meller H. Die neolithischen und bronzezeitlichen Goldfunde Mitteldeutschlands–Eine Übersicht. In: Meller H, Risch R, Pernicka E, editors. Metalle der Macht ‐ Frühes Gold und Silber. 6. Mitteldeutscher Archäologentag vom 17. bis 19. Oktober 2013 in Halle (Saale). Tagungen des Landesmuseums für Vorgeschichte Halle 11. Halle: Landesmuseum für Vorgeschichte Halle (Saale); 2014. p. 611–716.

[55] Gömöri J, Melis E, Kiss V. A cemetery of the Gáta–Wieselburg culture at Nagycenk. Acta Archaeologica Academiae Scientiarum Hungaricae. 2018;69:5–82.

[56] Novotná M. Die Nadeln in der Slowakei. Prähistorische Bronzefunde XIII(6). München: C.H. Beck; 1980.

[57] Gedl M. Die Nadeln in Polen I (Frühe und ältere Bronzezeit). Prähistorische Bronzefunde XIII(7). München: C.H. Beck; 1983.

[58] Ch Horn. Studien zu den europäischen Stabdolchen. Universitätsforschungen zur prähistorischen Archäologie 246. Bonn: Rudolf Habelt GmbH; 2014.

[59] Ernée M. Prag-Miškovice. Archäologische und naturwissenschaftliche Untersuchungen zu Grabbau, Bestattungssitten und Inventaren einer frühbronzezeitlichen Nekropole. Römisch-Germanische Forschungen72. Darmstadt: Philipp von Zabern; 2015.

[60] Stockhammer PW, Massy K, Knipper C, Friedrich R, Kromer B, Lindauer S, et al. Rewriting the Central European Early Bronze Age Chronology: Evidence from Large-Scale Radiocarbon Dating. PLoS ONE. 2015;10(10): e0139705. doi: 10.1371/journal.pone.0139705 26488413 PMC4619067

[61] Sarnowska W. Kultura unietycka w Polsce. Vol. I. Wrocław-Warszawa-Kraków: Ossolineum; 1969.

[62] Knoll F, Meller H. Die Ösenkopfnadeln–ein „Klassen” verbindenes Trachtelement der Aunjetitzer Kultur. Ein Beitrag zu Kontext, Interpretation und Typochronologie der mitteldeutschen Exemplare. In: Meller H, Hahn HP, Jung R, Risch R, editors. Arm und Reich–Zur Ressourcenverteilung in prähistorischen Gesellschaften. 8. Mitteldeutscher Archäologentag vom 22. bis 24. Oktober 2015 in Halle (Saale). Tagungen des Landesmuseums für Vorgeschichte Halle 14. Halle: Landesmuseum für Vorgeschichte Halle (Saale); 2016. p. 283–370.

[63] Stróżyk M. Pejzaż z kurhanami. Krajobraz funeralny społeczności kręgu kultur mogiłowych na pograniczu śląsko-wielkopolskim. Bibliotheca Fontes Archaeologici Posnanienses 23. Poznań: Muzeum Archeologiczne w Poznaniu; 2019.

[64] Czebreszuk J. Schyłek neolitu i początki epoki brązu w strefie południowo-zachodniobałtyckiej. Poznań: Wydawnictwo Naukowe UAM; 2001.

[65] Schwenzer S. Przysieka Polska–Ein Grabfund in der Ungebung der frühbronzezeitlichen Siedlung in Bruszczewo/Przysieka Polska–znalezisko grobowe w sąsiedztwie osady w Bruszczewie. In: Czebreszuk J, Müller J, editors. Ausgrabungen und Forschungen in einer prähistorischen Siedlungskammer Großpolens/Badania mikroregionu osadniczego z terenu Wielkopolski. Band I/ Część I. Forschungsstand–Erste Ergebnisse–Das östliche Feuchtbodenareal/Stan badań –Pierwsze wyniki–Wschodnia, torfowa część stanowiska. Poznań-Kiel-Rahden/Westf.: Verlag Marie Leidorf GmbH; 2004. p. 317–342.

[66] Zich B. Aunjetitzer Herrschaften in Mitteldeutschland– „Fürsten”der Frühbronzezeit und ihre Territorien („Domänen“). In: Meller H, Hahn HP, Jung R, Risch R, editors. Arm und Reich–Zur Ressourcenverteilung in prähistorischen Gesellschaften. Rich and Poor–Competing for resources in prehistoric societies. 8. Mitteldeutscher Archäologentag vom 22. bis 24. Oktober 2015 in Halle (Saale). Tagungen des Landesmuseums für Vorgeschichte Halle 14. Halle: Landesmuseum Halle; 2016. p. 371–406.

[67] Princes Meller H., Gold Weapons and Armies. Reflections on the Dieskau gold find and its possible origin from the Early Bronze Age Bornhöck barrow near Dieskau in the Saalekreis district. Studia Hercynia. 2020;XXIII(2):9–21.

[68] Meller H. Princes, Armies, Sanctuaries: The Emergence of Complex Authority in the Central German Únětice Culture. Acta Archaeologica. 2019;90(1):39–79.

[69] Becker B, Jäger K-D, Kaufmann D, Litt T. Dendrochronologische Datierungen von Eichenhölzern aus den frübronzezeitlichen Hügelgräbern bei Helmsdorf und Leubingen (Aunietizer Kultur) und an bronzezeitlichen Fluβeichen bei Merseburg. Jahresschrift für mitteldeutsche Vorgeschichte. 1989;72:299–312.

[70] Furmanek M, Lasak I. Początki wczesnej epoki brązu na Śląsku w świetle aktualnych badań nad chronologią. Śląskie Sprawozdania Archeologiczne. 2013; LV:5–23.

[71] Pokutta DA. Population Dynamics, Diet and Migrations of the Unetice Culture in Poland. GOTARC Series B. No. 60. Gothenburg Archaeological Theses. Gothenburg: University of Gothenburg Press; 2013.

[72] Nicklisch N, Ramsthaler F, Bunnefeld JH, Schulz G, Friedrich R, Alt KW, Meller H. Bioarchaeological investigations of the princely grave at Helmsdorf attesting to the violent death of an Early Bronze Age leader. Scientific Reports. 2002;12:16139. 10.1038/s41598-022-20720-8 PMC9515160 36168035

[73] Müller J, Kneisel J. Bruszczewo: Production, distribution, consumption and the formation of social differences, in: Müller J, Czebreszuk J, Kneisel J, editors. Bruszczewo II. Badania mikroregionu osadniczego z terenu Wiekopolski/Ausgrabungen und Forschungen in einer prähistorischen Siedlungskammer Großpolens. Studien zur Archäologie in Ostmitteleuropa/Studia nad Pradziejami Europy Środkowej 6. Bonn: Verlag Dr. Rudolf Habelt GmbH; 2010. p. 756–783.

[74] Dörfler W, Feeser I, Hildebrandt-Radke I, Rzodkiewicz M. Environment and settlement ‐ A multiproxy record of holocene palaeoenvironmental development from Lake Wonieścć, Greater Poland. Vegetation History and Archaeobotany. 2022;32(2):1–18.

[75] Czebreszuk J. The Bronze Age in the Polish Lands. In: Fokkens H, Harding A, editors. The Oxford Handbook of the European Bronze Age. Oxford: Oxford University Press; 2013. p. 767–786.

[76] Otto KH. Die sozioökonomischen Verhältnisse bei den Stämmen der Leubinger Kultur in Mitteldeutschland. Ethnographische-Archäologische Forschungen 3. Berlin: Deutscher Verlag der Wissenschaften; 1955.

[77] Steffen C. Die Prunkgräber der Wessex- und Aunjetitz-Kultur. BAR International Series 2160. Oxford: Archaeopress; 2010.

[78] Müller J. Radiocarbonchronologie–Keramiktechnologie–Osteologie–Anthropologie–Raumanalysen. Beiträg zum Neolithikum und Frühbronzezeit im Mittelelbe-Saale-Gebiet. Bericht der Römisch-Germanischen Kommission 80. Mainz am Rhein: Zabern; 1999.

[79] Czebreszuk J. At the confluence of the Corded Ware and the Únětice cultures. The case of amber discs. Studia Hercynia 2022;XXVI(1):32–48.

[80] Pospieszny Ł. Zwyczaje pogrzebowe społeczności kultury ceramiki sznurowej w Wielkopolsce i na Kujawach. Poznań; 2009.

[81] Größler H. Das Fürstengrab im großen Galgenhügel am Paulsschachte bei Helmsdorf (im Mansfelder Seekreis). Jahresschrift für die Vorgeschichte der Sächsisch-Thüringischen Länder. 1907;6:1–87.

[82] Ernée M.: Jantar v české únětické kultuře–k počátkům jantarové stezky. Památky archeologické. 2012;CIII:71–172

[83] Czebreszuk J. Bursztyn w kulturze mykeńskiej. Zarys problematyki badawczej. Poznań: Wydawnictwo Naukowe UAM; 2011.

